# Impact of chronic kidney disease on adults and their caregivers in the United States: a systematic literature review

**DOI:** 10.1186/s12882-026-04847-8

**Published:** 2026-03-02

**Authors:** Katherine M. Osenenko, Satabdi Chatterjee, Shelagh M. Szabo, Bonnie M. K. Donato

**Affiliations:** 1 Broadstreet HEOR, 300 - 177 West 7th Ave, Vancouver, BC V5Y 1L8 Canada; 2 Boehringer Ingelheim Pharmaceuticals, Inc., Ridgefield, CT USA

**Keywords:** Chronic kidney disease, Patient burden, Caregiver burden, Systematic review

## Abstract

**Background:**

The objective of this study was to synthesize estimates of the economic and humanistic burden among adults with chronic kidney disease (CKD) and the economic, humanistic, and clinical impacts among their informal caregivers, in the United States (US).

**Methods:**

A systematic literature review was conducted using MEDLINE and Embase, to identify studies reporting estimates of (1) economic or humanistic burden among adults with CKD and (2) economic, humanistic, or clinical impacts on their informal caregivers, published between 2016 and 2025. Two reviewers independently performed study selection and data extraction according to PRISMA guidelines. Study population characteristics were summarized, as were estimates of burden; costs were converted to 2025 USD.

**Results:**

*Adults with CKD*: From 10,191 citations, 55 studies were included. Mean age ranged from 46 to 81 years; 30–73% were female. Out-of-pocket (OOP) expenditures were estimated at $1,619, $2,189 (mean), and $1,913 (median) annually (3 studies). Health-related quality of life (HRQoL) was assessed in 48 studies using a variety of instruments. Substantial HRQoL impacts were consistently demonstrated, reported by adults with CKD; higher CKD stages and presence of diabetes were associated with greater impacts. *Caregivers*: From 5,909 citations, 7 studies were included. Caregivers were primarily female (64–100%); 24–70% were the care recipient’s spouse/partner; and mean age ranged from 47 to 59 years. Time spent caregiving ranged from 27 to 38 h/week (2 studies) and caregiving duration was approximately 4 years (3 studies). Depression and anxiety were reported in 21–36% and 32–44% of caregivers, respectively (2 studies). Although various instruments were used to measure caregiver burden and HRQoL, studies consistently documented the high impact on caregivers (6 studies).

**Conclusion:**

This review demonstrates the considerable burden experienced by adults with CKD and their informal caregivers, and highlights several gaps in characterizing these impacts, particularly for economic burden among adults with CKD, and economic and humanistic burden among caregivers. Further research is needed to better describe and quantify these impacts, as well as evaluate modifiable risk factors that contribute to greater burden.

**Supplementary Information:**

The online version contains supplementary material available at 10.1186/s12882-026-04847-8.

## Introduction

Chronic kidney disease (CKD) is one of the most common non-communicable diseases worldwide, and its prevalence is increasing [1]. In the United States (US), it is estimated that 35.5 million individuals (or 14% of the US general population) have CKD [1, 2], and that by the year 2030, 16.7% of US adults aged 30 years and older will be affected [3]. Among US adults with type 2 diabetes mellitus (T2DM), hypertension and obesity, rates of CKD are higher than among adults without these conditions, estimated at 39%, 30%, and 17% [1], respectively. Further, CKD disproportionately affects certain racial and ethnic minority populations: African American, Native American, and Hispanic adults are all more likely to experience kidney failure compared to their white counterparts [4]. CKD is classified into five stages, based on estimated glomerular filtration rate and kidney function, ranging from mild kidney damage (stage 1) to kidney failure requiring dialysis or transplant (stage 5) [5]. 

CKD is associated with substantial clinical, economic, and humanistic burden. Individuals with CKD have an increased risk of experiencing cardiovascular disease (CVD) [6], progression to kidney failure [6, 7] and death. In the US, approximately 85,000 deaths are attributed to CKD annually [2], making CKD the tenth leading cause of death [2]. In 2019, Medicare spending for CKD and end-stage kidney disease (ESKD) reached $87.2 billion and $37.2 billion, respectively [8]. The economic burden associated with CKD has been shown to increase with the presence of comorbidities, in particular T2DM, and with each progressive CKD stage [9]. 

Beyond the burden to healthcare systems and payors, adults with CKD and those who provide them with informal care are also impacted, both from an economic and humanistic perspective. Adults with CKD may be faced with out-of-pocket (OOP) expenditures and lost productivity due to their condition, as well as considerable impact on their health-related quality of life (HRQoL). Additionally, adults with CKD often rely upon the support of unpaid informal caregivers who play a critical role in assisting with disease management and care, activities of daily living, as well as providing psychosocial and emotional support [10]. A 2020 national survey reported that 47.9 million (19.2%) US adults provided informal unpaid care for another adult, a significant increase from 2015 [11]. Among these, 89% provided care for a family member and 40% lived with the care recipient [11]. Informal caregivers often experience challenges in maintaining their own health and well-being, and reported impacts include physical, emotional and financial strain. Notably, caregivers of adults with CKD may experience increased rates of psychological distress, mental health diagnoses, fatigue, sleep disturbances, and negative HRQoL impacts, in particular when caring for those in advanced CKD stages [10, 12, 13]. The nature and extent of the impacts to informal caregivers may be influenced by both caregiver-related factors (e.g., demographics, nature of caregiving role, relationship to care recipient, health status) and care recipient factors (e.g., disease severity, health status and functional limitations, level of dependence on caregiver) [14, 15]. 

While the burden to healthcare systems and payors is well-characterized, less information has been published on the impact to adults with CKD and those who provide them with informal care, considering both the economic and humanistic impacts. Further, there is a corresponding lack of published data on the factors that may influence these impacts among both adults with CKD and their caregivers, such as CKD stage or presence of comorbid T2DM among those with CKD.

## Objectives

The primary objective was to synthesize estimates of the economic and humanistic burden among adults with CKD; and the associated economic, humanistic, and clinical impacts among their informal caregivers. Secondary objectives were to synthesize these estimates by CKD stage and by the presence of T2DM, as well as to identify key drivers of these effects among adults with CKD and their informal caregivers.

## Methods

### Study identification

A systematic literature review (SLR) was conducted to identify published estimates of the economic and humanistic burden among those with CKD, and the associated economic, humanistic, and clinical burden among caregivers, followed by a synthesis of available evidence from the previous nine years. This review followed the Preferred Reporting Items for Systematic Reviews and Meta-Analyses (PRISMA) statement [16], and was guided by the pre-specified PICOS (Population, Intervention, Comparator, Outcome, Study design) criteria (Table 1).

**Table 1:** Scope of literature review

Criteria	Adults with CKD burden	Caregiver burden
Population	Adults with CKD in the US • Any stage, including end-stage kidney disease (ESKD) and kidney transplant • With or without comorbid T2DM	Caregivers for adults with CKD in the US • Any stage, including ESKD and kidney transplant • With or without comorbid T2DM
Intervention/ comparators	Any medications, or none^1^	
Outcomes	Population characteristics; including but not limited to:^2^ • Age • Sex • Ethnicity/race • Geographical location • Employment status • Income • Insurance type • Comorbidities (T2DM, CVD of particular interest) • Access to caregiver support (Yes/No) Economic outcomes (including by stages of CKD) • Direct costs to adults with CKD (e.g., out-of-pocket expenses)^3^ • Indirect costs • Time missed from work / absenteeism • Lost productivity / presenteeism • Disability Humanistic outcomes (including by stages of CKD) • Standardized measures of burden^4^ • Standardized measures of well-being^4^ • Standardized measures of HRQoL^4^ Qualitative • Drivers of burden^2^	Population characteristics of caregivers; including but not limited to:^2^ • Age • Sex • Ethnicity/race • Geographical location • Employment status • Income • Insurance type • Clinical burden, provided by comorbidities (anxiety, depression, stress, other mental health) Population characteristics of adults with CKD being provided care; including but not limited to:^2^ • Age • Sex • Ethnicity/race • CKD stage • Comorbidities (T2DM, CVD of particular interest) Economic outcomes (including by stages of CKD) • Direct and indirect costs • Time spent caregiving • Lost productivity/presenteeism, time missed from work/absenteeism Humanistic outcomes • Standardized measures of caregiver burden^4^ • Standardized measures of well-being^4^ • Standardized measures of HRQoL^4^ Qualitative • Drivers of burden of caregiving^2^
Study design	Observational studies (prospective, cross-sectional, or retrospective)	

Abbreviations: CKD=chronic kidney disease; CVD=cardiovascular disease; HRQoL=health-related quality of life; US=United States; T2DM=type 2 diabetes mellitus

^1^ Studies focused on specific interventions, medications or devices were excluded

^2^ Extracted from papers reporting the economic or humanistic burden outcomes of interest for adults with CKD or caregivers for those with CKD

^3^ Direct medical costs for CKD were not included in the current review

^4^ Three most commonly-used, plus standardized generic measures

A comprehensive literature search using MEDLINE and Embase was initially performed on October 28, 2021 in Ovid^®^ to identify observational studies published in English during the preceding five years. Subsequently, the search was re-run on May 1, 2025 to identify additional studies published since the time of the original search. Customized search strategies – one each for the ‘adults with CKD burden search’ and ‘caregiver burden search’ were developed using a conceptual approach that included terms related to the population and outcomes (Supplemental Tables 1 and 2). Animal studies, non-US studies, letters to the editor, review articles, editorials, and commentaries were excluded. The search was limited to those published in the English language.

For each search, results were imported into EndNote (EndNote X8, Clarivate Analytics, Philadelphia PA); duplicate articles were removed. For the updated search (2025), duplicate citations identified in the original search (2021) were excluded. Two researchers independently screened abstracts and full texts to identify relevant articles. Discrepancies were discussed with a third senior-level researcher providing arbitration as needed. Studies were included if they met the PICOS criteria specified in Table 1. Peer reviewed articles published during or after 2016 and conference proceedings from the preceding two years (original search: 2019–2021; updated search: 2023–2025) that were identified from the search strategies were considered for inclusion. Conference proceedings were included to capture any recent studies that had not yet been published as peer-reviewed articles at the time the search was conducted. To focus on study populations most representative of those with CKD and cardiometabolic comorbidities, studies conducted among adults with CKD and T2DM were considered for inclusion, while studies conducted exclusively among those with type 1 diabetes were excluded. Studies were also considered for inclusion if they were conducted among a broad or unspecified ‘diabetes’ population. The systematic search was supplemented by hand searching the reference lists of review articles identified during the search, and from data identified from the grey literature.

### Data extraction

Double data extraction was performed for all study design, demographic, and outcomes data of interest from the eligible studies. For continuous variables, the mean, median, standard deviation, and range were extracted whenever available. For dichotomous and categorical variables, the number and proportion were extracted. Any discrepancies observed between the data extracted by the two data reviewers was resolved through discussion to achieve consensus. Double-data extraction of relevant parameters was performed using a customized Microsoft^®^ Excel^®^ workbook.

### Quality assessment

Included studies underwent a reporting quality assessment using the Strengthening the Reporting of Observational studies in Epidemiology (STROBE) statement [17]. The 22-item checklists scores studies based on a scale ranging from 0 (low quality) to 22 (high quality) (Supplemental Table 3).

### Data synthesis

Summary tables were created along with a narrative description of the data and methods used in the eligible studies. Multiple publications reporting on the same study were considered as a single study for the SLR, with the most recent data summarized in the SLR. Key outcomes of all studies were reported in study-level tables, and for outcomes reported across multiple studies, ranges, means, and medians were described. Costs were converted to 2025 US dollars (USD) using US healthcare specific consumer price index (CPI) values. If the original study did not report the cost year, the publication year was used for inflating costs.

## Results

**Fig. 1:**
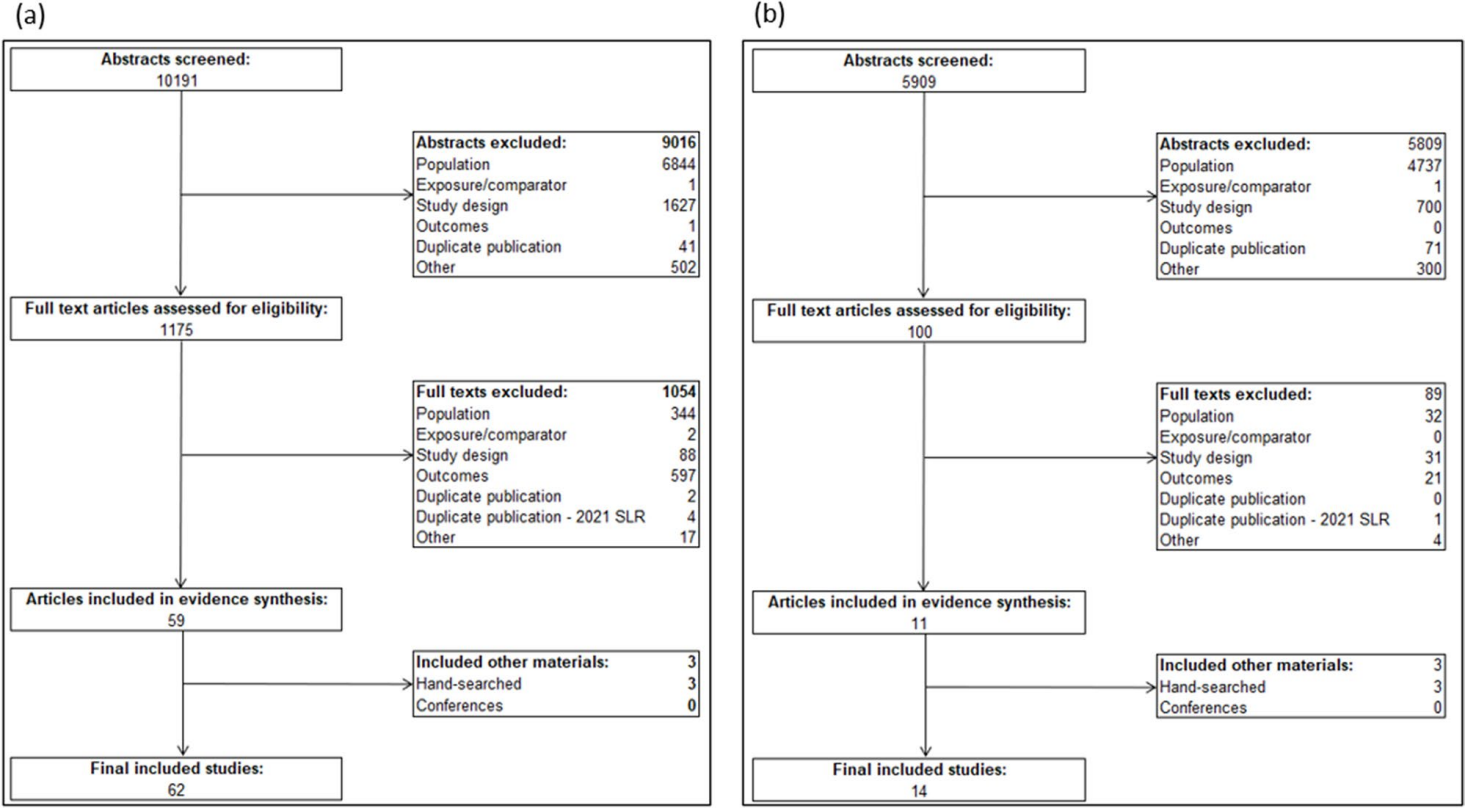
PRISMA flow diagram depicting study inclusion/exclusion for (**a**) adults with CKD burden SLR; (**b**) caregiver burden SLR

From 10,191 citations identified in the adults with CKD burden search, 1175 were eligible for full-text review and 59 were included in the synthesis (Fig. 1a). An additional 3 citations were identified by hand searching and included full-text articles of abstracts already identified for inclusion. The 62 included articles and abstracts corresponded to 55 unique studies. From 5,909 citations identified in the caregiver burden search, 100 were eligible for full-text review, and 11 were included in the synthesis (Fig. 1b). An additional 3 citations were identified by hand searching and included full-text articles of abstracts identified for inclusion. The 14 included articles and abstracts corresponded to 7 unique studies.

### Quality assessment

Using the STROBE criteria, the majority of included studies for the adults with CKD burden component (*n* = 40; 72.7%) had a score of 85% or higher (≥ 19 items of the 22 items on the STROBE checklist; Supplemental Table 4), indicating high quality.

For the caregiver burden component, 4 studies (57.1%) had a score of 85% or higher. Studies rated as lower quality tended to be congress abstracts.

### Study and study population characteristics

#### Adults with CKD

Of the 55 included studies, 11 reported on the economic outcomes of interest and 48 reported on the humanistic outcomes of interest (Supplemental Table 5). Eighteen studies were cross-sectional, 27 were prospective and 10 used retrospective observational study designs. The number of adults with CKD included ranged from 10 [18] to 240,343 [19]. 

Study populations included those with CKD (*n* = 17 studies), those with kidney failure / undergoing dialysis (28 studies) and those who were pre-/post-transplant (10 studies; Supplemental Table 6). Mean age ranged from 46.3 years [20] to 80.5 years [21], and the proportion of females ranged from 30% [22] to 72.5% [23]. Common comorbidities included T2DM (23.0 [24]-80.2% [25]), CVD (12.0 [26]-41.5% [27]) and hypertension (45.5 [24]-96.8% [28]) Four studies included populations consisting entirely of adults with both T2DM and kidney disease [29–32]. Several studies reported the frequency of comorbid depression, which ranged from 15.0% [33] to 32.6% [34] among those with CKD. One study reported that more than half of adults with CKD described receiving paid or unpaid care related to their health condition (CKD plus anemia: 58.9%; CKD - no anemia: 50.9%) [35]. The overall average number of care hours received weekly was 14.2 in the anemia cohort and 11.3 h in the no anemia cohort [34]. 

#### Caregivers

Of the seven included studies, 4 were cross-sectional and 3 were prospective observational study designs (Supplemental Table 7). The number of caregivers included ranged from 10 [18] to 258. %) [35]. One study included caregivers and adults with CKD, with and without anemia [35], and a second study included caregiver-care recipient dyads among kidney transplant recipients [18]. Another study of caregivers to adults with CKD evaluated six months of participation in a peer-led mentoring program, either face-to-face or online [36]. Among all seven studies, there were very limited data reported on the characteristics of those for whom care was being provided (Supplemental Table 8). Study populations included those with CKD (3 studies), those with kidney failure undergoing dialysis (2 studies), and kidney transplant recipients (1 study); one study included those with kidney failure undergoing dialysis as well as those who were post-kidney transplant. One study reported that more than 70% of adults with CKD receiving care were aged 55 years or older, more than half (53.5%) were male, and the majority (72.5%) were Caucasian [35]. None of the included studies reported CKD stage for the care recipients. One study reported that 35.8% (CKD – no anemia) to 50.9% (CKD plus anemia) of the care recipients had comorbid diabetes [35]. 

### Economic burden

#### Adults with CKD – direct costs

Three studies reported OOP expenditures among adults with CKD, using data from the Medical Expenditure Panel Survey (MEPS) [24, 27, 37]. Mean OOP expenditures in 2010–2011 were estimated to be $1,619 annually among adults with CKD; and were $1,579 (privately insured), $1,620 (publicly insured), and $2,088 (uninsured) annually (CPI-adjusted to 2025 USD; Table 2) [24]. More recently, mean OOP expenditures in 2018–2021 were estimated to be $2,189 annually among adults with CKD [37]. It was estimated that 12.9% of adults with CKD experienced high OOP burden in 2011, which was defined as family OOP healthcare spending exceeding 10% of family income (Table 3) [24]. Among those who were privately insured, publicly insured, or uninsured, high OOP burden was experienced by 7.2%, 17.2% and 27.0%, respectively, in 2011 [24]. Median (IQR) annual OOP expenditures (2011–2013) were estimated at $1,913 ($937-$3,719) among adults with non-dialysis dependent kidney disease) [36]. 

**Table 2:** Direct costs among adults with CKD

Author, Year	Population description	Subgroup / cost type	*n*	Annual cost [mean (SD)] (2025 USD)
Ozieh 2019 [24]	Kidney disease, aged 18–64 yrs (MEPS data,^1, 2^ 2010-2011)	Overall	616	$1,619
		Privately insured	316	$1,579
		Publicly insured	201	$1,620
		Uninsured	80	$2,088
Small 2017 [36]	Non-dialysis dependent kidney disease; aged ≥ 21 yrs (MEPS data,^1^ 2011–2013)	-	52	Median (IQR): $1,913 ($937-$3,719)
Das 2024 [37]	CKD (MEPS data,^1^ 2018–2021)	Total costs *(calculated)*	141	$2,189
		Inpatient stay		$241
		Emergency department visits		$140
		Hospital outpatient visits		$147
		Office-based visits		$856
		Prescription medicine costs		$805

Abbreviations: CKD=chronic kidney disease; IQR=interquartile range; MEPS=Medical Expenditure Panel Survey; N=number; SD=standard deviation; USD=United States dollars

Notes:

^1^ Out-of-pocket expenditures. MEPS OOP expenditures include self-reported coinsurance and deductible payments plus cash outlays for supplies, services and items not covered by health insurance. Health insurance premiums are not included

^2^ Most recent years in the study period for which data are reported

**Table 3:** Measures of financial burden or hardship among adults with CKD

Author, Year	Population description	Population subgroup	*n*	Outcome description	Experiencing burden/hardship *n* (%)
Acquah 2021 [38]	CKD; aged 18–64 yrs (NHIS data)	Overall	1,425	No financial hardship^2^	758 (53.2)
				Financial hardship but able to pay^2^	342 (24.0)
				Unable to pay at all^2^	325 (22.8)
		Privately insured	546	No financial hardship^2^	283 (51.8)
				Financial hardship but able to pay^2^	179 (32.8)
				Unable to pay at all^2^	84 (15.4)
		Medicaid	554	No financial hardship^2^	344 (62.1)
				Financial hardship but able to pay^2^	87 (15.7)
				Unable to pay at all^2^	123 (22.2)
		Uninsured	185	No financial hardship^2^	44 (23.8)
				Financial hardship but able to pay^2^	47 (25.4)
				Unable to pay at all^2^	94 (50.8)
		Other	139	No financial hardship^2^	87 (62.6)
				Financial hardship but able to pay^2^	29 (20.9)
				Unable to pay at all^2^	23 (16.5)
Kim 2024 [23]	Kidney failure	Overall	40	Financial toxicity^3^	11 (27.5)
Ozieh 2019 [24]	Kidney disease, aged 18–64 yrs (MEPS data, 2011)^1^	Overall	616	High OOP burden^4^	75 (12.1)
		Privately insured	316	High OOP burden^4^	23 (7.2)
		Publicly insured	201	High OOP burden^4^	35 (17.2)
		Uninsured	80	High OOP burden^4^	22 (27.0)

Abbreviations: CKD=chronic kidney disease; MEPS=Medical Expenditure Panel Survey; N=number; NHIS=National Health Interview Survey; OOP=out-of-pocket

Notes:

^1^ Most recent year in the study period for which data are reported

^2^ Financial hardship due to medical bills

^3^ Financial burden specifically related to the cost of treatment

^4^ High OOP burden was defined as family OOP healthcare spending exceeding 10% of family income

One study reported that 46.9% of non-elderly adults with CKD (National Health interview Survey [NHIS]) experienced financial hardship within the past year due to medical bills (Table 3), including those that were able to pay their medical bills (24.0%) and those that were unable to pay at all (22.8%) [38]. Lack of health insurance was the strongest determinant of financial hardship due to medical bills, and reported impacts included non-adherence and delayed/foregone medical care.

Another study evaluated financial distress using the In Charge Financial Distress/Financial Well-being 8-item Scale with responses ranging from 1 (high distress) to 10 (low/no distress) [39]. The mean (SD) score among the sample of African American adults on a kidney transplant wait list was 6.2 (2), indicating moderate financial distress (*data not shown*). A third study reported financial toxicity, defined as financial burden specifically related to the cost of treatment, among 27.5% of those with kidney failure [23]. 

#### Adults with CKD – indirect costs

Three studies reported on activity impairment and lost productivity using the Work Productivity and Activity Impairment (WPAI)-Specific Health Problem instrument (Table 4) [35]. Average impairment in nonwork-related activities ranged from 20.6% (CKD) [40] to 55.5% (CKD – dialysis-dependent) [41]. In one study, the average impairment in nonwork-related activities was higher among those with anemia: 51.9% (CKD plus anemia) and 39.2% (CKD – no anemia). For employed adults, reductions in work productivity ranged from 15.6% (CKD) to 44.9% (CKD plus anemia). In one study, 52.6% of employed adults with CKD plus anemia and 48.2% of employed adults with CKD - no anemia, made at least one work-related decision due to their condition, including reducing work hours, taking a leave of absence, retiring early or quitting their job (Table 5) [35]. Among those with CKD plus anemia and CKD – no anemia, the most common work-related accommodations were to retire early (20.5% and 22.7%, respectively), quit their employment (19.5% and 15.9%), and reduce work hours (20.0% and 14.1%) [35]. 

**Table 4:** Activity and work impairment among adults with CKD^1^

Author, Year	Impairment description	Population description	*N*	Level of impairment (%)^3^
Michalopoulos 2022 [34, 35]	Activity impairment^2^	CKD plus anemia	190	51.9
		CKD - no anemia	220	39.2
	Overall work impairment	CKD plus anemia	55	44.9
		CKD - no anemia	78	35.4
	Work time missed (absenteeism)	CKD plus anemia	55	14.7
		CKD - no anemia	78	13.7
	Impairment while working (presenteeism)	CKD plus anemia	55	37.9
		CKD - no anemia	78	28.9
Chadban 2023 [41] *(abstract)*	Activity impairment^2^	CKD – dialysis-dependent	64	55.5
		CKD – non-dialysis-dependent	135	43.7
	Work time missed (absenteeism)	CKD – dialysis-dependent	64	21.6
		CKD – non-dialysis-dependent	135	9.6
Pollock 2025 [40]	Activity impairment^2^	CKD	174	20.6
	Overall work impairment			15.6
	Work time missed (absenteeism)			6.4
	Impairment while working (presenteeism)			13.5

Abbreviations: CKD=chronic kidney disease; N=number

Notes:

^1^ Only one study reported activity and work impairment

^2^ Activity impairment = the impact of caregiving on the ability to complete daily activities

^3^ Higher percentages indicate greater impairment

**Table 5:** Work-related impacts among adults with CKD^1^

Author, Year	Description	*N*	Work-related decision	Number making decision *n* (%)
Michalopoulos 2022 [34, 35]	CKD plus anemia pts	55	At least 1 work-related decision	52.6
			Retired earlier than planned	20.5
			Decreased the number of hours worked per week	20.0
			Quit job or employment	19.5
			Took a leave of absence	10.0
			Changed jobs or employers	8.9
			Declined a job advancement (transfer or promotion)	5.8
			Changed shift	4.7
			Increased the number of hours worked per week	0.0
			None	44.7
	CKD - no anemia pts	78	At least 1 work-related decision	48.2
			Retired earlier than planned	22.7
			Decreased the number of hours worked per week	14.1
			Quit job or employment	15.9
			Took a leave of absence	8.2
			Changed jobs or employers	8.2
			Declined a job advancement (transfer or promotion)	5.0
			Changed shift	5.0
			Increased the number of hours worked per week	2.3
			None	49.1

Abbreviations: CKD=chronic kidney disease; N=number

Notes:

^1^ Only one study reported job-related decisions due to caregiving

One study evaluated whether pre-ESKD nephrology clinical care (> 6 months pre-dialysis) had an impact on employment (i.e., to help those with CKD remain employed) [42]. At the time of dialysis initiation, 49% of adults with CKD reported receiving pre-ESKD nephrology clinical care and 62% were employed; however, pre-ESKD care 6 months before dialysis initiation was not associated with remaining employed [42]. 

None of the included studies reported direct or indirect costs to adults with CKD by stage or by the presence of comorbid T2DM.

#### Caregivers – indirect costs

The duration of caregiving was reported in 3 studies and the time spent caregiving was reported in 2 studies. The impact of caregiving on caregiver work productivity was reported in two publications [35, 41]. Among the 3 studies that described the duration of caregiving, the mean (SD) ranged from 3.5 (5.1) to 4.1 (5.6) years (Table 6). Two publications described the mean (SD) hours spent caregiving per week, which ranged from 27 (27) hours per week (dialysis care recipients; African American female caregivers) [43] to 38 (41) hours per week (CKD care recipients, no anemia) [35]. Further, in the study of caregivers for adults with CKD with and without anemia, the mean (SD) weekly hours spent caregiving increased to 52.6 (42.5) and 42.8 (41.6), respectively, among the subset of caregivers who lived with the care recipient (50.9% and 60.8% of caregivers, respectively; *data not shown in table*) [35]. 

**Table 6:** Duration of caregiving and time spent caregiving, among caregivers of adults with CKD

Author, Year	Caregiver description (Care recipient description)	*N*	Mean duration of caregiving (SD), years	Mean time spent caregiving per week (SD), hours
Affinito et al., 2018 [44]	Primary unpaid CG; aged ≥ 18 years (Dialysis)	89	4.1 (5.6)	NR
Ghahramani et al., 2022 [36, 45]	CG; aged ≥ 18 years; undergoing peer-led mentoring	86	NR	NR
Michalopoulos 2022 [34, 35]	CG; aged ≥ 18 years (CKD)	258	NR	NR
	CG; aged ≥ 18 years (CKD plus anemia)	110	3.5 (5.1)	33.6 (35.8)
	CG; aged ≥ 18 years (CKD – no anemia)	148	3.7 (3.9)	38.0 (40.9)
Rasmussen et al., 2020 [46]	CG, spouse or partner (All)	99	NR	NR
	CG, spouse or partner (Pre-transplant)	65	NR	NR
	CG, spouse or partner (Post-transplant)	34	NR	NR
Starks, 2019 [43]	CG, aged ≥ 18 years, African American women (Dialysis)	75	4.1 (4.6)	27 (27)

Abbreviations: CG=caregiver; CKD=chronic kidney disease; N=number; NR=not reported; SD=standard deviation

Two studies reported on activity impairment and lost productivity, using the WPAI-Caregiver instrument (Table 7) [35, 41]. In one study, caregivers of dialysis dependent adults with CKD reported higher rates of both impairment in non-work-related activities and work absenteeism (44.3% and 14.8%, respectively) compared to caregivers of non-dialysis dependent adults with CKD (34.3% and 10.5%, respectively) [41]. In a second study, among all caregivers (employed and unemployed), the average impairment in nonwork-related activities was 49.0% (CKD plus anemia, care recipients) and [41] 42.9% (CKD - no anemia, care recipients) [34]. For employed caregivers, corresponding reductions in work productivity were 47.9% (CKD plus anemia, care recipients) and 40.7% (CKD - no anemia, care recipients) (Table 7) [35]. In this same study, 69.1% of employed caregivers for those with CKD plus anemia and 53.4% of employed caregivers for those with CKD - no anemia, made at least one work-related decision due to caregiving and the patient’s health, including reducing work hours, taking a leave of absence, retiring early or quitting their job (Table 8) [35]. Among caregivers to those with CKD plus anemia and CKD – no anemia, the most common work-related decision by caregivers was to reduce work hours (31.8% and 17.6%, respectively); the frequency of other work-related decisions varied between the two caregiver groups [35]. 

**Table 7:** Activity and work impairment among caregivers of adults with CKD^1^

Author, Year	Impairment description	Caregiver description (Care recipient description)	*N*	Level of impairment (%)^3^
Michalopoulos 2022 [34, 35]	Activity impairment due to caregiving^2^	CG; aged ≥ 18 years (CKD plus anemia)	110	49.0
		CG; aged ≥ 18 years (CKD - no anemia)	148	42.9
	Overall work impairment due to caregiving	Employed CG; aged ≥ 18 years (CKD plus anemia)	67	47.9
		Employed CG; aged ≥ 18 years (CKD - no anemia)	74	40.7
	Work time missed due to caregiving (absenteeism)	Employed CG; aged ≥ 18 years (CKD plus anemia)	67	19.0
		Employed CG; aged ≥ 18 years (CKD - no anemia)	74	14.8
	Impairment while working due to caregiving (presenteeism)	Employed CG; aged ≥ 18 years (CKD plus anemia)	67	37.9
		Employed CG; aged ≥ 18 years (CKD - no anemia)	74	33.2
Chadban 2023 [41] *(abstract)*	Activity impairment due to caregiving^2^	CG; aged ≥ 18 years (CKD – dialysis dependent)	NR^4^	44.3
		CG; aged ≥ 18 years (CKD – non-dialysis dependent)	NR^4^	34.3
	Work time missed due to caregiving (absenteeism)	CG; aged ≥ 18 years (CKD – dialysis dependent)	NR^4^	14.8
		CG; aged ≥ 18 years (CKD – non-dialysis dependent)	NR^4^	10.6

Abbreviations: CG=caregiver; CKD=chronic kidney disease; N=number; NR=not reported

Notes:

^1^ Only one study reported activity and work impairment

^2^ Activity impairment = the impact of caregiving on the ability to complete daily activities

^3^ Higher percentages indicate greater impairment

^4^ Study included 113 caregivers and 199 adults with CKD; among those with CKD, 32.2% were dialysis dependent

**Table 8:** Work-related decisions due to caregiving, among caregivers of adults with CKD^1^

Author, Year	Caregiver description (Care recipient description)	*N*	Work-related decision due to caregiving	Number making decision *n* (%)
Michalopoulos 2022 [34, 35]	Employed CG; aged ≥ 18 years (CKD plus anemia)	67	At least 1 work-related decision	69.1
			Reduced hours worked per week	31.8
			Leave of absence	20.9
			Changed shift	19.1
			Changed jobs/employers	19.1
			Quit job/employment	16.4
			Declined job advancement	11.8
			Early retirement	8.2
			Increased hours worked per week	7.3
			None	30.0
	Employed CG; aged ≥ 18 years (CKD - no anemia)	74	At least 1 work-related decision	53.4
			Reduced hours worked per week	17.6
			Leave of absence	6.8
			Changed shift	11.5
			Changed jobs/employers	9.5
			Quit job/employment	11.5
			Declined job advancement	8.1
			Early retirement	12.2
			Increased hours worked per week	3.4
			None	43.9

Abbreviations: CG=caregiver; CKD=chronic kidney disease; N=number

Notes: ^1^ Only one study reported job-related decisions due to caregiving

None of the included studies reported indirect caregiver costs by CKD stage or the presence of comorbid T2DM among CKD care recipients.

### Humanistic burden

#### Adults with CKD - HRQoL

The majority of included studies reported on HRQoL among adults with CKD, rather than on economic outcomes or measures of burden or well-being. The most frequently used instrument to assess HRQoL was the Kidney Disease and Quality of Life-36 survey (KDQOL-36; 17 studies). Eight studies used the KDQOL-SF, which is a different version of the KDQOL-36. A variety of generic instruments were also used to assess HRQoL, including the SF-12, SF-36, and EQ-5D.

**Fig. 2:**
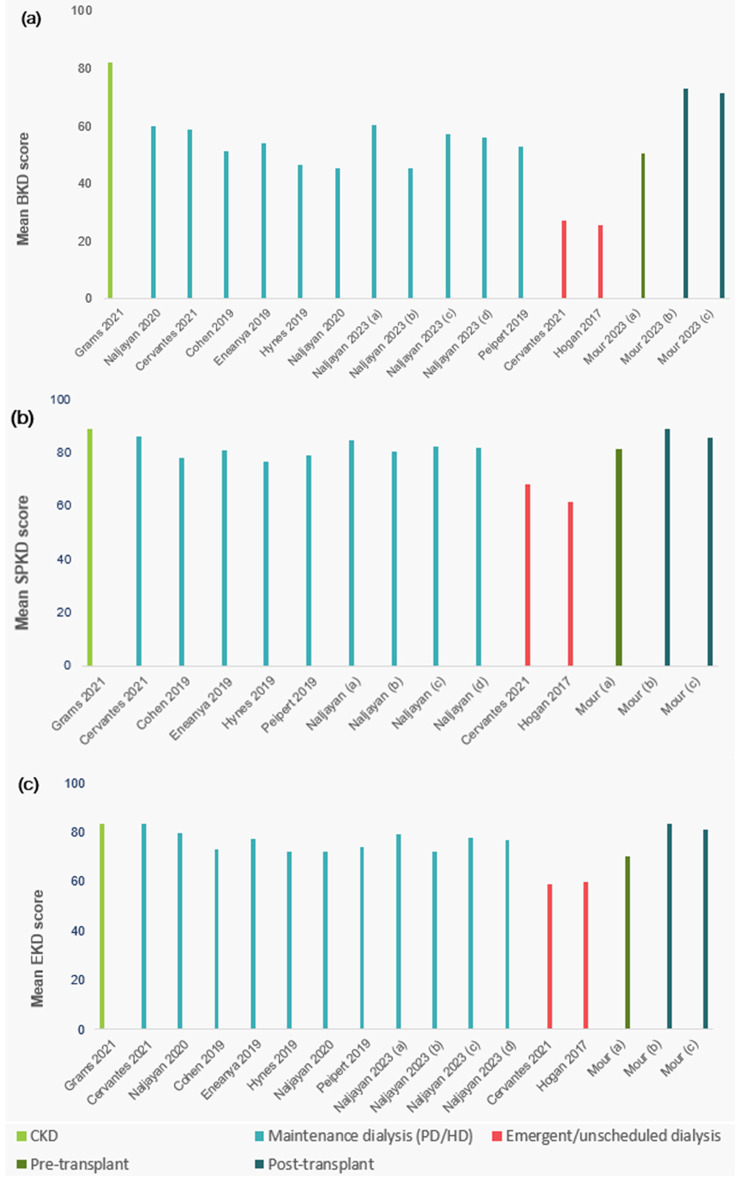
Disease-specific HRQOL among adults with CKD, from studies using KDQOL-36: (**a**) Burden of Kidney Disease subscale scores; (**b**) Symptoms and Problems of Kidney Disease subscale scores; (**c**) Effects of Kidney Disease subscale scores. Notes: Decreasing scores indicate lower health and functioning (greater burden, symptoms, effects). 1) Naljayan 2023; (a) Incremental CAPD, (b) Full CAPD, (c) Incremental APD, (d) full APD. 2) Mour 2023; (a) KT waitlist; (b) 4 months post-KT, (c) 1 year post-KT. Abbreviations: APD=automated peritoneal dialysis; BKD=Burden of Kidney Disease subscale; CAPD=continuous ambulatory peritoneal dialysis; CKD=chronic kidney disease; EKD=Effects of Kidney Disease subscale; HD=hemodialysis; HRQoL=health-related quality of life; KDQOL-36 = Kidney Disease and Quality of Life-36 survey; KT=kidney transplant; PD=peritoneal dialysis; SPKD=Symptoms and Problems of Kidney Disease subscale

For the 17 studies that used the KDQOL-36, mean scores for the Burden of Kidney Disease, Symptoms and Problems of Kidney Disease, and Effects of Kidney Disease subscales, as well as the SF-12 Mental Component Summary and Physical Component Summary scores, are summarized in Supplemental Table 10. Higher scores on each of the disease-specific subscales indicate better health and functioning. Mean (SD) burden subscale scores ranged from 11.35 (4.43) among adults with kidney failure to 82.2 (23.7) among adults with CKD in the Chronic Renal Insufficiency Cohort (i.e., greater health and functioning). In another study of adults undergoing emergent/unscheduled hemodialysis, burden scores increased from 27.4 (19.5) to 59.1 (33.6) once maintenance dialysis was initiated [47]. Among studies reporting burden of kidney disease scores, increasing burden, symptoms/problems and effects of kidney disease were observed with increase in disease severity and the need for dialysis (Fig. 2). Similarly, one study reported burden, symptoms/problems, and effects of kidney disease scores by CKD stage and presence of T2DM, with all subscale scores generally worsening with increasing disease stage and presence of diabetes) [48]. Mean (SD) burden of kidney disease scores ranged from 71.2 (27.8; CKD stages 4–5 plus diabetes) to 88.8 (19.2; CKD stage 3a; symptoms/problems of kidney disease scores from 79.6 (15.4; CKD stage 3b, plus diabetes) to 87.1 (13.2; CKD stage 3a, no diabetes); and effects of kidney disease scores from 83.1 (18.5; CKD stage 4–5 plus diabetes) to 93.3 (12.5; G3b, no diabetes) [48]. 

**Fig. 3:**
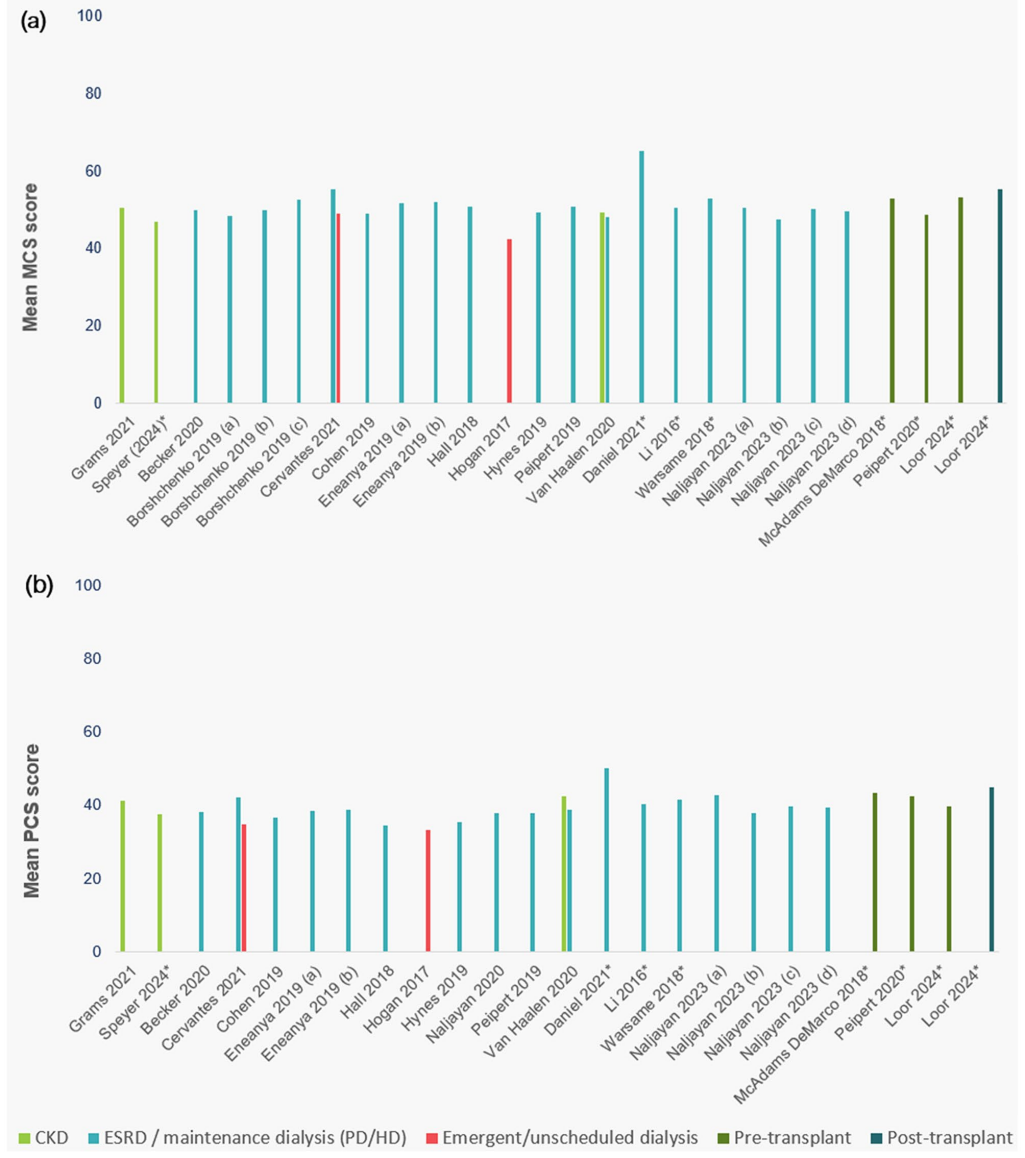
SF-12 mental component summary (**a**) and physical component summary (**b**) scores among adults with CKD, from studies using KDQoL-36 and KDQoL-SF. *Studies used KDQoL-SF; all other studies used KDQoL-36 instrument. Notes: 1) Borshchenko included adults who traveled to their HD appointments by (a) ambulance, (b) taxi, or (c) self/family ride. 2) Eneanya 2019 included adults receiving dialysis, on (**a**) day 0-120 after initiation and (**b**) day 365–485 after initiation). 3) Naljayan 2023; (a) Incremental CAPD, (b) Full CAPD, (c) Incremental APD, (d) full APD. 4) Mour 2023; (a) KT waitlist; (b) 4 months post-KT, (c) 1 year post-KT. Abbreviations: APD=automated peritoneal dialysis; CAPD=continuous ambulatory peritoneal dialysis; CKD=chronic kidney disease; HD=hemodialysis; HRQoL=health-related quality of life; KDQOL-36 = Kidney Disease and Quality of Life Short-Form survey; KT=kidney transplant; MCS=mental component summary; PCS=physical component summary; PD=peritoneal dialysis

Among studies that used the KDQOL-SF, the majority only reported SF-12 mental and physical component subscale scores (Supplemental Table 11). Mean mental and physical subscale scores from studies using either the KDQoL-36 or the KDQoL-SF are presented in Fig. 3. One study reported both the EQ-5D visual analogue scale and the SF-12 mental and physical subscale scores by CKD stage; with HRQoL scores generally decreasing with increasing disease stage (Supplemental Table 12) [49]. 

#### Caregivers - burden

Six of the seven included studies reported on at least one measure of caregiver burden or HRQoL. Of the burden scales, the Zarit Burden Inventory (ZBI) was the most commonly used (3 studies). Other burden scales included the Burden Scale for Family Caregivers-Short Version (BSFC-s) and the Caregiver Stress Appraisal (CSA) instruments, which were each described in one study. Additionally, one study included the Positive Aspects of Caregiving (PAC) instrument. The ZBI is 22-item assessment originally designed to quantify burden among caregivers of elderly adults with dementia [50]. ZBI items are rated on a scale of zero (never) to four (nearly always), with total overall scores ranging from 0 to 88; higher scores are indicative of greater burden. Of three studies using the ZBI, two reported the mean score and one reported the median (Supplemental Table 13). Mean (SD) caregiver burden scores ranged from 14.0 (7.8) among caregivers who had undergone 18 months of face-to-face peer mentoring to 23.6 (12.1) among caregivers before participation in a peer mentoring program (online group) [36]. Median (IQR) scores ranged from 16 (12–24) among caregivers of post-kidney transplant recipients, to 19 (11–35) among caregivers of pre-kidney transplant recipients. Caregivers had lower burden scores after participation in peer-mentoring programs and after the care recipient had undergone kidney transplant.

The BSFC-s is a validated instrument measuring the burden of caregiving on a 0 to 30 scale. Scores are interpreted as: 0–4: no to low burden; 5–14: moderate burden; 15–30: severe-to-very severe burden [51]. Corresponding risk levels of experiencing physical psychosomatic complaints are no increased risk, increased risk, and very much increased risk, respectively. One study reported BSFC-s scores for a sample of caregivers for adults with CKD with and without anemia (Supplemental Table 14) [35]. The majority of caregivers reported severe-to-very severe burden due to caregiving, with estimates ranging from 59% to 76% of caregivers. Overall, a greater percentage of those caring for adults with CKD plus anemia experienced severe-to-very-severe burden (69%), compared to those caring for adults with CKD - no anemia (59%). This was consistent and more pronounced among the subset of caregivers living with the care recipient (76% vs. 59%, respectively).

The CSA scale measures caregiver stress and burden on a 12-item scale; scores range from 12 to 48, with higher scores representing greater burden experienced by the caregiver [52]. In one study of 89 caregivers of adults with renal failure on hemodialysis, mean (SD) burden score was 29.4 (9.7), indicating moderate stress and burden among caregivers; scores ranged from 12 to 48 across all caregivers [44]. 

The PAC scale is a nine-item instrument assessing perceived benefits associated with caregiving; scores range from 9 to 45, with higher scores indicating more positive feelings about their caregiving experience [53]. One study reported PAC scores among a sample of 89 caregivers to adults with renal failure on hemodialysis [44]. Mean (SD) score was 36.7 (6.6) and scores ranged from 9 to 45 across sample of caregivers.

#### Caregivers - HRQoL

Three studies included a measure of HRQoL; two reported SF-12 scores [18, 46] and the other reported Quality of Life Index (QLI) scores [43]. The SF-12 measures HRQoL impact in eight domains, with each item scored on a scale of 0-100 and higher scores indicating better health. Scores are often divided into the Mental Component Score and the Physical Component Score [54]. Among 99 caregivers of pre-transplant recipients on dialysis and post-transplant recipients, the overall median (IQR) SF-12 score was 45 (39–50) [46]. Caregivers of post-transplant recipients had higher median SF-12 scores (49 [42–52]) than caregivers of pre-transplant recipients (44 [38–48]; *p* = 0.03). In a small longitudinal study of kidney transplant caregiver-recipient dyads (*n* = 10), median caregiver SF-12 mental and physical summary scores did not differ significantly in the 12 months following kidney transplantation (mental: baseline = 49 [42–56], 12 months = 50 [45–55], *p* = 0.94; physical: baseline = 54 [47–61], 12 months = 52 [44–60], *p* = 0.51) [18]. 

The QLI is a 33-item instrument that measures QoL and life satisfaction, with scores ranging from 0 to 30 and higher scores reflecting a more favorable rating [55]. Among 75 African American women caregivers of dialysis recipients, mean (SD) QLI score was 16.6 (1.4) [43]. Lower QLI scores were associated with a greater number of hours worked at paid employment (*p* < 0.001), and higher QLI scores associated with more hours of care provided (*p* = 0.003) and the care recipient being of older age (*p*=0.001). Higher QLI scores were associated with providing care to a child, rather than for a sibling, husband, or parent, as well as with living with the care recipient.

None of the included studies reported caregiver burden or HRQoL impacts by CKD stage or the presence of comorbid T2DM among CKD care recipients.

## Discussion

The substantial clinical and economic burden associated with CKD is well-established; however, less is known about the economic and humanistic impacts among adults with CKD or their informal caregivers, particularly in the US. Those with CKD face high OOP expenses, lost productivity, and impairments to their HRQOL, all of which grow higher in the presence of comorbid conditions. Further, the level of care required by adults with patients may be demanding for caregivers, in turn affecting caregivers’ own health and well-being. A thorough characterization of the impact on adults with CKD and their informal caregivers is important to fully understand the burden associated with CKD. This systematic literature review was conducted to synthesize estimates of the economic and humanistic burden among adults with CKD and their informal caregivers in the US.

The findings from this review demonstrate the considerable burden experienced by adults with CKD as well as by their informal caregivers, and highlight gaps in the evidence base. For those with CKD, although multiple disease-specific HRQoL estimates were identified, estimates of direct costs to adults with CKD, indirect costs, and lost productivity were sparse. Further, few studies reported data by CKD stage or presence of comorbid T2DM. Additionally, although several included studies consisted entirely of adults with both T2DM and kidney disease, direct comparison to other studies was limited. Mean OOP expenditures among adults with CKD were approximately $2,200 USD annually in the most recent study [37], and several studies reported that a lack of health insurance was associated with greater OOP burden and financial hardship, which may contribute to non-adherence to treatments and delayed/foregone medical care [24, 38]. Indeed, a systematic review on the impact of OOP costs in CKD reported that OOP costs were associated with early discontinuation from treatment programs, poorer adherence, and decreased dialysis sessions, across different geographical regions; lower income households and uninsured individuals were disproportionately affected [56]. Substantial impairments to work productivity due to both absenteeism and presenteeism were reported for employed adults with CKD, with one study estimating reductions of up to 45% in work productivity and that approximately half of adults with CKD make job-related changes to accommodate their CKD, such as reducing work hours or taking a leave of absence [35]. Similar impacts have been observed in other countries, with one study conducted in the UK, Mexico, Germany and the US reporting that CKD negatively impacted work productivity scores by up to 31% across all countries. Further, those with CKD had substantially worse financial well-being compared to the general population [57]. 

Published data on the impacts to caregivers of those with CKD were very limited: only seven eligible studies were identified. Very limited data were available on care recipient characteristics, and available estimates of caregiver burden were not summarized by care recipient CKD stage or presence of comorbid T2DM. None of the included publications reported direct costs to caregivers associated with caring for adults with CKD, such as OOP costs paid by the caregiver. Nonetheless, studies included in this review described an extensive time commitment, substantial impacts to productivity, and the high level of burden experienced by caregivers. Living with the care recipient was associated with greater hours spent caregiving [35]. While employed and unemployed caregivers may be impacted differently by the demands of caregiving, nonetheless substantial impairments to both overall activity impairment and work productivity were demonstrated. Among employed caregivers these included both absenteeism and presenteeism, with caregivers experiencing reductions of more than 40% in work productivity in one study; and more than half of caregivers also reported making at least one job-related change due to caregiving [35]. Across two studies, caregivers experienced reductions of more than 34% in non-work related activities [34, 41]. One study reported higher rates of impairment in non-work related activities and in work absenteeism among caregivers of dialysis-dependent adults compared to caregivers of non-dialysis-dependent adults with CKD [41]. Although data on lost productivity among caregivers to those with CKD are limited, these findings are consistent with other studies showing the increased impacts to dialysis-dependent adults with CKD and their caregivers [10, 12, 13]. 

Adults with CKD and their caregivers experience substantial impacts to HRQoL. Disease-specific scales were most often used to assess the impact on HRQoL among adults with CKD. In particular, considerable impacts in the burden subscale of the KDQOL-36 were observed, which increased with increasing disease stage. Similar increases in burden with increasing disease stage were also seen with the EQ-5D visual analogue scale. There was an overall lack of data on HRQoL impacts among adults with CKD and comorbid conditions such as T2DM, hypertension, or CVD; however, one study reported greater impacts on all KDQOL subscales with increase CKD stage and presence of T2DM [48]. 

Several different scales were used to assess caregiver burden and HRQoL. Caregiver burden scores from the ZBI were fairly consistent across studies and indicated moderate to high caregiver burden [36, 43, 46]. One study reported statistically significant reductions in burden scores among caregivers who participated in peer mentoring programs [36]. Notably, in one study using the BSFC-s scale, almost 60% of caregivers experienced severe-to-very severe burden [35]. Despite the substantial burden, caregivers also experienced positive perceptions and feelings about their caregiving role [44]. A recent systematic review on the burden among caregivers to adults with ESKD found that caregivers may experience both positive and negative impacts of caregiving, influenced by a variety of patient and caregiver-related factors [14, 15]. Although a protocol was recently published for an SLR to assess measurement properties of instruments used to assess caregiver burden in advanced kidney disease, published findings are not yet available. This research may provide insights into the optimal instruments for assessing caregiver burden in future research [58]. 

Caregiver HRQoL was assessed using the SF-12 in two studies. The overall median score of 45 reported in one study indicates lower than average HRQoL, compared to the general population norm of 50; [43, 46] however, mental component and physical component scores were not reported separately [46]. A second study reported caregiver mental component scores of 45 to 50 and physical component scores of 47 to 54, in the year following kidney transplant, indicating greater mental than physical HRQoL impacts; however, this study was conducted among a small number of caregivers (*n* = 10) [18]. Further, both studies were conducted among caregivers to dialysis and kidney transplant recipients, who may experience different HRQoL impacts than those with less severe disease. A recent international study reported that caregivers to dialysis-dependent adults with CKD experienced greater negative impairment to care-related HRQoL than caregivers to non-dialysis dependent adults with CKD [59]. Among a sample of female caregivers to dialysis recipients, factors associated with lower HRQoL included higher education levels, being employed full-time or part-time, and higher caregiver burden [46]. The impact to caregivers, both positive and negative, may be affected by many factors and will vary between individuals. The relationship between caregiver and care recipient is one such factor and there is the potential that closer relationships and living together may be associated with both increased burden and positive perceptions of the caregiving role [35, 44], which may also impact HRQoL.

This review used rigorous systematic review methodology to identify estimates of the economic and humanistic burden among adults with CKD, as well as the economic, humanistic, and clinical impacts among their informal caregivers in the US. Although numerous observational studies were identified that reported on the HRQoL impacts to adults with CKD, limited data were identified on the economic impacts to adults with CKD and the economic and humanistic impacts to their informal caregivers. Still, the rigorous and comprehensive nature of the systematic review methodology provides certainty that these areas represent a gap in the literature. Although a small number of caregiver studies were identified, all but one reported relatively large sample sizes of greater than 50 caregivers. These studies also represented various geographic regions across the US.

As with all evidence syntheses, this review was limited by the validity and quality of the included studies. Although the number of included studies was small for some outcomes of interest, multiple aspects of burden were considered (i.e., economic and humanistic), and included studies described at least one of these aspects. In addition, the outcome assessments described in the included publications varied, as well as the study populations. As a result, it was not possible to synthesize estimates of burden in a meaningful way based on the outcomes reported. Further, the comparability of adults with CKD and caregiver populations across different studies was unclear. Characteristics of adults with CKD such as insurance status, employment, income, and comorbidities were not consistently reported between studies. Such data can aid in the comparison of study findings and may be valuable for understanding the baseline burden experienced by adults with CKD as well as their caregivers.

Caregiver characteristics such as overall health status, financial status, and availability of social support may be valuable for understanding the baseline level of burden experienced by caregivers, as caregivers with greater comorbidities, worse financial status, and lower social support are likely to have a higher baseline burden. However, these types of caregiver data were sparse, such that the burden reported across different studies may not be meaningfully compared and interpreted even if estimates were available to be synthesized. Further to this, there was variability in the care recipient populations and few data reported on their characteristics, which limited comparability between studies as well as the assessment of drivers of burden. Few studies reported data by CKD stage or presence of comorbid T2DM, which limited the ability to address the secondary study objectives. Finally, as this review focused on US-specific estimates, findings may not be generalizable to other geographic regions.

Data from this synthesis highlight the substantial economic and humanistic burden among adults with CKD and their informal caregivers in the US, although data were limited on the drivers of burden and the magnitude of their impact. Still, available estimates demonstrate increased burden with advancing disease stage and presence of comorbidities. As such, clinical management strategies and treatments that slow CKD progression and reduce the burden of comorbidities may be critical to reducing the economic and humanistic impact among adults with CKD and their caregivers. With the availability of newer therapies that can delay CKD progression, additional research would be key to better understanding how these new therapies might help to alleviate burden among those with CKD and their informal caregivers.

## Conclusion

This review demonstrates the considerable burden experienced by adults with CKD and their informal caregivers in the US, and also highlights the data gap that exists in fully characterizing these impacts. In particular, data were more limited on the HRQoL impacts among caregivers and the economic outcomes among both adults with CKD and their caregivers. Despite limited economic burden data, notable findings included the substantial financial burden and lost productivity that may be faced by adults with CKD and the considerable impacts to their HRQoL, as well as the substantial amount of time spent caregiving, the substantial impact to productivity, and the high burden experienced by caregivers to those with CKD. Few studies assessed the drivers of burden among adults with CKD and their caregivers, including the impacts of disease stage and presence of comorbid T2DM. Future work will be important to better characterize the economic burden to adults with CKD and their informal caregivers, including the indirect costs and lost productivity, the humanistic burden among caregivers, as well as the evaluation of modifiable risk factors that contribute to greater burden and potential impacts of new therapies that delay CKD progression.

## Supplementary Information

Below is the link to the electronic supplementary material.

Supplementary Material 1

## Data Availability

The datasets used and/or analysed during the current study are available from the corresponding author on reasonable request.

## Abbreviations

BKD: Burden of Kidney Disease
BSFC-s: Burden Scale for Family Caregivers-Short Version
CAPD: Continuous ambulatory peritoneal dialysis
CG: Caregiver
CPI: Consumer price index
CSA: Caregiver Stress Appraisal
CVD: Cardiovascular disease
EKD: Effects of Kidney Disease subscale
ESKD: End-stage kidney disease
HD: Hemodialysis
HRQOL: Health-related quality of life
IQR: Interquartile range
KDQOL: Kidney Disease and Quality of Life
KT: Kidney transplant
MEPS: Medical Expenditure Panel Survey
NHIS: National Health interview Survey
NR: Not reported
PAC: Positive Aspects of Caregiving
PCS: Physical component summary
PD: Peritoneal dialysis
PICOS: Population, Intervention, Comparator, Outcome, Study design
QLI: Quality of Life Index
SD: Standard deviation
SLR: Systematic literature review
SPKD: Symptoms and Problems of Kidney Disease subscale
STROBE: Strengthening the Reporting of Observational studies in Epidemiology
WPAI: Work Productivity and Activity Impairment
ZBI: Zarit Burden Inventory
