# Supplementary material for: Impact of chronic kidney disease on adults and their caregivers in the United States: a systematic literature review
Source: BMC Nephrol. 2026 Mar 2;27:218. doi: 10.1186/s12882-026-04847-8 (PMC13059210; doi:10.1186/s12882-026-04847-8)
Supplement: Supplementary file 1 — Supplementary Material 1 [file 12882_2026_4847_MOESM1_ESM.pdf]

Supplemental content

Supplemental Table 1. Search strategy for patient impact SLR

#	Search term	Number of hits (October 2021)	Number of hits (May 2025)
1	Kidney Diseases/ or Diabetic Nephropathies/	163423	317312
2	exp Renal Replacement Therapy/	426890	505690
3	Renal Insufficiency/	75003	186989
4	exp Renal Insufficiency, Chronic/	240251	382521
5	(dialysis or hemodialysis or haemodialysis or hemofiltration or haemofiltration or hemodiafiltration or haemodiafiltration or kidney disease or renal disease or kidney failure or renal failure or ESRF or ESKF or ESRD or ESKD or CKF or CKD or CRF or CRD or CAPD or CCPD or APD or predialysis or pre-dialysis or diabetic nephropath* or diabetic kidney or DKD).tw.	904607	1117538
6	((kidney or renal) adj (transplant* or graft* or allograft*)).tw.	231240	272140
7	or/1-6	1307977	1695671
8	exp "Costs and Cost Analysis"/	620066	713496
9	exp Absenteeism/	27756	31242
10	exp Presenteeism/	2182	3664
11	value of life/	148006	181653
12	"quality of life"/	752657	1027757
13	"cost of illness"/	50013	56588
14	patient reported outcome measures/	41665	91188
15	quality-adjusted life years/	44003	57867
16	sickness impact profile/	9631	9727
17	activities of daily living/	146662	205590
18	(economic* or pharmaco?economic* or cost*).ti.	416759	507798
19	(price* or pricing* or budget* or financ* or fee or fees or productivity or absenteeism or presenteeism or sickness impact profile or disability adjusted life or qal* or qtime* or qwb* or daly* or euroqol* or eq5d* or eq 5d* or qol* or hql* or hqol* or h qol* or hrqol* or hr qol* or health utility* or utility score* or disutilit* or hui or hui1 or hui2 or hui3 or health* year* equivalent* or hye or hyes or rosser or willingness to pay or time?trade?off or tto or standard gamble* or adl or patient reported outcome* or patient-reported outcome* or sf?36 or short?form?36 or sf?20 or short?form?20 or sf?12 or short?form?12 or sf?8 or short?form?8 or sf?6 or short?form?6).ti,ab.	954732	1317598
20	(value adj2 (money or monetary)).ti,ab.	6270	7893
21	(quality adj2 (wellbeing or well-being)).ti,ab.	5465	8313
22	((economic or humanistic) adj2 burden).ti,ab.	35049	51559
23	or/8-22	2428099	3169492
24	exp United States/	2690245	2960403
25	(united states or USA or "U.S.A." or "U.S." or America*).ti,ab,jw,in.	19135984	22416193
26	24 or 25	20217779	23539674
27	letter/	2281740	2579930

#	Search term	Number of hits (October 2021)	Number of hits (May 2025)
28	editorial/	1267776	1530947
29	news/	209833	230036
30	exp historical article/	405656	415840
31	anecdotes as topic/	47892	53112
32	comment/	935520	1050391
33	(letter or comment*).ti.	385730	468479
34	animals/ not humans/	5863125	6548738
35	(exp africa/ or exp antarctic regions/ or exp arctic regions/ or exp asia/ or exp oceania/ or europe/)	3160217	3879092
36	or/27-35	13160925	15151964
37	7 and 23 and 26	23390	35271
38	37 not 36	20687	31161
39*	38 use ppezv	5820	7076
40	exp renal replacement therapy/	426890	505690
41	kidney disease/ or chronic kidney disease/ or kidney failure/ or chronic kidney failure/ or stage 1 kidney disease/ or mild renal impairment/ or moderate renal impairment/ or severe renal impairment/ or end stage renal disease/ or renal replacement therapy-dependent renal disease/ or kidney transplantation/ or diabetic nephropathies/	848382	1057261
42	(dialysis or hemodialysis or haemodialysis or hemofiltration or haemofiltration or hemodiafiltration or haemodiafiltration or kidney disease or renal disease or kidney failure or renal failure or ESRF or ESKF or ESRD or ESKD or CKF or CKD or CRF or CRD or CAPD or CCPD or APD or predialysis or pre-dialysis or diabetic nephropath* or diabetic kidney or DKD).tw.	904607	1117538
43	((kidney or renal) adj (transplant* or graft* or allograft*)).tw.	231240	272140
44	or/40-43	1408184	1724154
45	exp "cost of illness"/	50013	59466
46	exp "opportunity cost"/	424	1107
47	exp cost/	620066	713496
48	exp productivity/	79659	93135
49	exp absenteeism/	27756	31242
50	exp presenteeism/	2182	3664
51	exp health care cost/	378631	442438
52	exp daily life activity/	98980	128782
53	quality adjusted life year/	44003	57867
54	"quality of life index"/	2926	3428
55	"quality of life"/	752657	1027757
56	"cost of illness"/	50013	56588
57	patient-reported outcome/	44302	91188
58	short form 12/	7712	11532
59	short form 20/	149	207
60	short form 36/	33157	45049
61	short form 8/	513	748

#	Search term	Number of hits (October 2021)	Number of hits (May 2025)
62	sickness impact profile/	9631	9727
63	or/18-22,45-62	2325232	3030267
64	exp United States/	2690245	2960403
65	(united states or USA or "U.S.A." or "U.S." or America*).ti,ab,jx,in.	17886400	21066159
66	64 or 65	19061840	22286167
67	letter.pt. or letter/	2359307	2669402
68	note.pt.	870118	1006760
69	editorial.pt.	1291207	1559088
70	(letter or comment*).ti.	385730	468479
71	animal/ not human/	5996498	6548738
72	or/67-71	10591574	11873327
73	44 and 63 and 66	25569	34407
74	73 not 72	23476	31719
75**	74 use oemezd	17964	24973
76***	39 or 75	23784	32049
77	limit 76 to english	23588	31834
78	limit 77 to conference abstract	10590	14567
79	limit 77 to conference paper	6408	7720
80	limit 77 to "conference review"	5779	7029
81	limit 77 to "review"	4757	5907
82	or/78-80	11259	15300
83	limit 82 to yr="2016 - 2018"	2427	1916
84	81 or 83	6997	7689
85	77 not 84	16591	24145
86	limit 85 to last 5 years	5565	NA
86	limit 85 to yr="2021-current"	NA	5848
87	remove duplicates from 86	**4880**	**5311**

* Medline (“ppezv”) results only
**Embase (“oemezd”) results only
***Combined MEDLINE and Embase results

The search strategy was run on October 28, 2021 in the following databases:

- Embase 1974 to October 27, 2021
- Ovid MEDLINE(R) and Epub Ahead of Print, In-Process & Other Non-Indexed Citations, Daily and Versions(R) 1946 to October 27, 2021

The search strategy was re-run on May 1, 2025 in the following databases:

- Embase 1974 to April 30, 2025
- Ovid MEDLINE(R) and Epub Ahead of Print, In-Process & Other Non-Indexed Citations, Daily and Versions(R) 1946 to April 30, 2025

Supplemental Table 2. Search strategy for caregiver impact SLR

#	Search term	Number of hits (October 2021)	Number of hits (May 2025)
1	Kidney Diseases/ or Diabetic Nephropathies/	163423	317312
2	exp Renal Replacement Therapy/	426890	505690
3	Renal Insufficiency/	75003	186989
4	exp Renal Insufficiency, Chronic/	240251	382521
5	(dialysis or hemodialysis or haemodialysis or hemofiltration or haemofiltration or hemodiafiltration or haemodiafiltration or kidney disease or renal disease or kidney failure or renal failure or ESRF or ESKF or ESRD or ESKD or CKF or CKD or CRF or CRD or CAPD or CCPD or APD or predialysis or pre-dialysis or diabetic nephropath* or diabetic kidney or DKD).tw.	903078	1117538
6	((kidney or renal) adj (transplant* or graft* or allograft*)).tw.	230977	272140
7	or/1-6	1305927	1695671
8	(caregiv* or 'care giver*' or caretaker* or carer* or partner* or 'next of kin' or family or families or parent* or spouse* or husband* or wife or wives or child or children or brother* or sister* or sibling* or friend* or 'social support' or 'social network*' or 'significant other*' or relative* or 'married person*' or 'spousal notification' or stepfamily or stepfamilies or 'support system*' or neighbo?r* or 'family caregiver*' or 'informal care').ti,ab.	9428767	11451995
9	Caregivers/	113697	192952
10	exp Social Support/	176243	221496
11	or/8-10	9498395	11539805
12	exp Stress, Psychological/	312136	397899
13	(burden or distress or stress or strain or overload or burnout).ti,ab.	3671093	4795800
14	exp "Costs and Cost Analysis"/	619279	713496
15	exp Absenteeism/	27740	31242
16	exp Presenteeism/	2175	3664
17	value of life/	147893	181653
18	"quality of life"/	750417	1027757
19	"cost of illness"/	49934	56588
20	patient reported outcome measures/	41340	91188
21	quality-adjusted life years/	43903	57867
22	sickness impact profile/	9628	9727
23	activities of daily living/	146364	205590
24	(economic* or pharmaco?economic* or cost*).ti.	416133	507798
25	(price* or pricing* or budget* or financ* or fee or fees or productivity or absenteeism or presenteeism or sickness impact profile or disability adjusted life or qal* or qtime* or qwb* or daly* or euroqol* or eq5d* or eq 5d* or qol* or hql* or hqol* or h qol* or hrqol* or hr qol* or health utility* or utility score* or disutilit* or hui or hui1 or hui2 or hui3 or health* year* equivalent* or hye or hyes or rosser or willingness to pay or time?trade?off or tto or standard gamble* or adl or patient reported outcome* or patient-reported outcome* or sf?36 or short?form?36 or sf?20 or short?form?20 or sf?12 or short?form?12 or sf?8 or short?form?8 or sf?6 or short?form?6).ti,ab.	952332	1317598
26	(value adj2 (money or monetary)).ti,ab.	6256	7893
27	(quality adj2 (wellbeing or well-being)).ti,ab.	5455	8313
28	((economic or humanistic) adj2 burden).ti,ab.	34942	51559

#	Search term	Number of hits (October 2021)	Number of hits (May 2025)
29	or/12-28	5910150	7666888
30	11 and 29	1198208	1579130
31	exp United States/	2687290	2960403
32	(united states or USA or "U.S.A." or "U.S." or America*).ti,ab,jw,in.	19112868	22416193
33	31 or 32	20194219	23539674
34	letter/	2279671	2579930
35	editorial/	1266104	1530947
36	news/	209637	230036
37	exp historical article/	405402	415840
38	anecdotes as topic/	47919	53112
39	comment/	933098	1050391
40	(letter or comment*).ti.	385138	468479
41	animals/ not humans/	5856839	6548738
42	(exp africa/ or exp antarctic regions/ or exp arctic regions/ or exp asia/ or exp oceania/ or europe/)	3153082	3879092
43	or/34-42	13143256	15151964
44	7 and 30 and 33	8944	13026
45	44 not 43	8049	11731
46	45 use ppezv	2223	2779
47	exp renal replacement therapy/	426066	505690
48	kidney disease/ or chronic kidney disease/ or kidney failure/ or chronic kidney failure/ or stage 1 kidney disease/ or mild renal impairment/ or moderate renal impairment/ or severe renal impairment/ or end stage renal disease/ or renal replacement therapy-dependent renal disease/ or kidney transplantation/ or diabetic nephropathies/	846972	1057261
49	(dialysis or hemodialysis or haemodialysis or hemofiltration or haemofiltration or hemodiafiltration or haemodiafiltration or kidney disease or renal disease or kidney failure or renal failure or ESRF or ESKF or ESRD or ESKD or CKF or CKD or CRF or CRD or CAPD or CCPD or APD or predialysis or pre-dialysis or diabetic nephropath* or diabetic kidney or DKD).tw.	903078	1117538
50	((kidney or renal) adj (transplant* or graft* or allograft*)).tw.	230977	272140
51	or/47-50	1406022	1724154
52	(caregiv* or 'care giver*' or caretaker* or carer* or partner* or 'next of kin' or family or families or parent* or spouse* or husband* or wife or wives or child or children or brother* or sister* or sibling* or friend* or 'social support' or 'social network*' or 'significant other*' or relative* or 'married person*' or 'spousal notification' or 'step family' or 'step families' or 'support system*' or neighbo?r* or 'family caregiver*' or 'informal care').ti,ab.	9428734	11451956
53	exp caregiver/	135787	193199
54	52 or 53	9442962	11470531
55	(burden or distress or stress or strain or overload or burnout).ti,ab.	3671093	4795800
56	burnout/	22030	33323
57	exp "cost of illness"/	49934	59466
58	exp "opportunity cost"/	417	1107
59	exp cost/	619279	713496
60	exp productivity/	79571	93135

#	Search term	Number of hits (October 2021)	Number of hits (May 2025)
61	exp absenteeism/	27740	31242
62	exp presenteeism/	2175	3664
63	exp health care cost/	378271	442438
64	exp daily life activity/	98859	128782
65	quality adjusted life year/	43903	57867
66	"quality of life index"/	2927	3428
67	"quality of life"/	750417	1027757
68	"cost of illness"/	49934	56588
69	patient-reported outcome/	43978	91188
70	short form 12/	7689	11532
71	short form 20/	148	207
72	short form 36/	33108	45049
73	short form 8/	510	748
74	sickness impact profile/	9628	9727
75	or/24-28,55-74	5734862	7446366
76	54 and 75	1119671	1479743
77	exp caregiver burden/	9119	13458
78	exp caregiver burnout/	439	1458
79	exp caregiver support/	3791	5496
80	or/76-79	1123032	1484492
81	exp United States/	2687290	2960403
82	(united states or USA or "U.S.A." or "U.S." or America*).ti,ab,jx,in.	17864259	21066159
83	81 or 82	19039147	22286167
84	letter.pt. or letter/	2357139	2669402
85	note.pt.	869443	1006760
86	editorial.pt.	1289426	1559088
87	(letter or comment*).ti.	385138	468479
88	animal/ not human/	5989752	6548738
89	or/84-88	10580122	11873327
90	51 and 80 and 83	9092	12415
91	90 not 89	8734	11965
92	91 use oemezd	6750	9413
93	46 or 92	8979	12192
94	limit 93 to english	8924	12134
95	limit 94 to last 5 years	3640	NA
95	limit 94 to yr="2021-current"	NA	3511
96	remove duplicates from 95	**2990**	**2919**

* Medline ("ppezv") results only
**Embase ("oemezd") results only
***Combined MEDLINE and Embase results

The search strategy was initially run on October 28, 2021 in the following databases:

- Embase 1974 to October 27, 2021

- Ovid MEDLINE(R) and Epub Ahead of Print, In-Process & Other Non-Indexed Citations, Daily and Versions(R) 1946 to October 27, 2021

The search strategy was re-run on May 1, 2025 in the following databases:

- Embase 1974 to April 30, 2025
- Ovid MEDLINE(R) and Epub Ahead of Print, In-Process & Other Non-Indexed Citations, Daily and Versions(R) 1946 to April 30, 2025
-

Supplemental Table 3. STROBE statement recommendation checklist

	Item No	Recommendation
Title and abstract	1	(a) Indicate the study's design with a commonly used term in the title or the abstract<br>(b) Provide in the abstract an informative and balanced summary of what was done and what was found
Introduction		
Background	2	Explain the scientific background and rationale for the investigation being reported
Objectives	3	State specific objectives, including any pre-specified hypotheses
Methods		
Study design	4	Present key elements of study design early in the paper
Setting	5	Describe the setting, locations, and relevant dates, including periods of recruitment,<br>exposure, follow-up, and data collection
Participants	6	(a) *Cohort study*—Give the eligibility criteria, and the sources and methods of selection of participants. Describe methods of follow-up<br>*Case-control study*—Give the eligibility criteria, and the sources and methods of<br>case ascertainment and control selection. Give the rationale for the choice of cases<br>and controls<br>*Cross-sectional study*—Give the eligibility criteria, and the sources and methods of<br>selection of participants<br><br>(b) *Cohort study*—For matched studies, give matching criteria and number of exposed and unexposed<br>*Case-control study*—For matched studies, give matching criteria and the number of<br>controls per case
Variables	7	Clearly define all outcomes, exposures, predictors, potential confounders, and effect<br>modifiers. Give diagnostic criteria, if applicable
Data sources/measurement	8	For each variable of interest, give sources of data and details of methods of assessment (measurement). Describe comparability of assessment methods if there<br>is more than one group
Bias	9	Describe any efforts to address potential sources of bias
Study size	10	Explain how the study size was arrived at

		Item No	Recommendation
	Quantitative variables	11	Explain how quantitative variables were handled in the analyses. If applicable, describe which groupings were chosen and why
	Statistical methods	12	(a) Describe all statistical methods, including those used to control for confounding<br>(b) Describe any methods used to examine subgroups and interactions<br>(c) Explain how missing data were addressed<br>(d) *Cohort study*—If applicable, explain how loss to follow-up was addressed<br>*Case-control study*—If applicable, explain how matching of cases and controls was addressed<br>*Cross-sectional study*—If applicable, describe analytical methods taking account of sampling strategy<br>(e) Describe any sensitivity analyses
Results			
	Participants	13	(a) Report numbers of individuals at each stage of study—e.g. numbers potentially eligible, examined for eligibility, confirmed eligible, included in the study, completing follow-up, and analyzed<br>(b) Give reasons for non-participation at each stage<br>(c) Consider use of a flow diagram
	Descriptive data	14	(a) Give characteristics of study participants (e.g. demographic, clinical, social) and information on exposures and potential confounders<br>(b) Indicate number of participants with missing data for each variable of interest<br>(c) Cohort study—Summarize follow-up time (e.g., average and total amount)
	Outcome data	15	*Cohort study*—Report numbers of outcome events or summary measures over time<br>*Case-control study*—Report numbers in each exposure category, or summary measures of exposure<br>*Cross-sectional study*—Report numbers of outcome events or summary measures
	Main results	16	(a) Give unadjusted estimates and, if applicable, confounder-adjusted estimates and their precision (e.g., 95% confidence interval). Make clear which confounders were adjusted for and why they were included<br>(b) Report category boundaries when continuous variables were categorized<br>(c) If relevant, consider translating estimates of relative risk into absolute risk for a meaningful time period
	Other analyses	17	Report other analyses done—e.g. analyses of subgroups and interactions, and sensitivity Analyses
Discussion			
	Key results	18	Summarize key results with reference to study objective
	Limitations	19	Discuss limitations of the study, taking into account sources of potential bias or imprecision.<br>Discuss both direction and magnitude of any potential bias
	Interpretation	20	Give a cautious overall interpretation of results considering objectives, limitations, multiplicity

	Item No	Recommendation
		of analyses, results from similar studies, and other relevant evidence
Generalizability	21	Discuss the generalizability (external validity) of the study results
Other Information		
Funding	22	Give the source of funding and the role of the funders for the present study and, if applicable, for the original study on which the present article is based

Supplemental Table 4. STROBE statement checklist for the included publications

Citation	STROBE Item Number																						Total number
	1	2	3	4	5	6	7	8	9	10	11	12	13	14	15	16	17	18	19	20	21	22	
**Caregiver burden SLR**																							
Affinito 2018	Y	Y	Y	Y	Y	Y	Y	Y	N	Y	Y	Y	Y	Y	Y	Y	N/A	Y	Y	Y	Y	N	19
Chadban 2023*	Y	Y	Y	Y	Y	Y	Y	Y	N	N	Y	N	N	Y	Y	N	N	Y	N	Y	N	Y	14
Ghahramani 2022	Y	Y	Y	Y	Y	Y	Y	Y	Y	Y	Y	Y	Y	Y	Y	Y	N/A	Y	Y	Y	Y	Y	21
Michalopoulos 2022	Y	Y	Y	Y	Y	Y	N	Y	N	Y	Y	Y	Y	Y	Y	Y	Y	Y	Y	Y	Y	Y	20
Rasmussen 2020	Y	Y	Y	Y	Y	Y	Y	Y	Y	Y	Y	Y	Y	Y	Y	Y	Y	Y	Y	Y	Y	Y	22
Starks 2019	N	Y	Y	Y	Y	Y	Y	Y	N	Y	Y	Y	Y	Y	Y	Y	N/A	Y	N	Y	Y	Y	18
Whittington 2025*	Y	Y	Y	Y	Y	Y	Y	Y	N	N	Y	N	N	Y	Y	N	N	Y	N	Y	N	Y	14
**Patient burden SLR**																							
Abedeen 2023*	Y	Y	Y	N	N	N	Y	N	N	N	N	N	N	Y	Y	N	NA	Y	N	N	N	NA	7
Acquah 2021	Y	Y	Y	Y	Y	Y	Y	Y	N	Y	Y	Y	Y	Y	Y	Y	N/A	Y	Y	Y	Y	Y	20
Adjeroh 2023	Y	Y	Y	Y	Y	Y	Y	Y	N	N	Y	Y	Y	Y	Y	Y	Y	Y	Y	Y	Y	Y	20
Awan 2020	Y	Y	Y	Y	Y	Y	Y	Y	Y	Y	Y	Y	Y	Y	Y	Y	Y	Y	Y	Y	Y	N	21
Becker 2020	Y	Y	Y	Y	Y	Y	Y	Y	Y	Y	Y	Y	Y	Y	Y	Y	N/A	Y	Y	Y	Y	Y	21
Becker 2022	Y	Y	Y	Y	Y	Y	Y	Y	Y	N	Y	Y	Y	Y	Y	Y	Y	Y	N	Y	N	Y	19
Borshchenko 2019*	Y	Y	Y	Y	Y	Y	Y	Y	N	N	Y	N	N	Y	Y	N	N	Y	N	Y	N	Y	14
Brown 2021	Y	Y	Y	Y	Y	N	Y	Y	Y	Y	Y	Y	Y	Y	Y	Y	Y	Y	Y	Y	Y	Y	21
Cabacungan 2019*	Y	Y	Y	Y	Y	Y	N	N	N	N	N	N	N	Y	Y	Y	N	Y	N	Y	N	N	11
Cervantes 2021	Y	Y	Y	Y	Y	Y	Y	Y	N	Y	Y	Y	Y	Y	Y	Y	N	Y	Y	Y	Y	Y	20
Chadban 2023*	Y	Y	Y	Y	Y	Y	Y	Y	N	N	Y	N	N	Y	Y	N	N	Y	N	Y	N	Y	14
Cohen 2019	Y	Y	Y	Y	Y	Y	Y	Y	N	Y	Y	Y	Y	Y	Y	Y	N/A	Y	Y	Y	Y	Y	20
Daniel 2021	Y	Y	Y	Y	N	Y	Y	Y	N	Y	Y	Y	N	Y	Y	Y	Y	Y	Y	Y	Y	Y	19
Das 2024*	Y	Y	Y	Y	Y	Y	Y	N	N	Y	N	N	Y	Y	Y	Y	NA	Y	N	Y	N	N	14
Debnath 2021	Y	Y	Y	Y	N	Y	Y	Y	N	Y	Y	Y	Y	Y	Y	Y	N/A	Y	Y	Y	Y	Y	19
Domenick Sridharan 2018	Y	Y	Y	Y	Y	Y	Y	Y	N	Y	Y	Y	Y	Y	Y	Y	Y	Y	Y	Y	Y	Y	21
Egeolu 2023	Y	Y	Y	Y	Y	Y	Y	Y	Y	N	Y	Y	N	Y	Y	Y	NA	Y	N	Y	N	NA	16
Eneanya 2019	Y	Y	Y	Y	Y	Y	Y	Y	N	Y	Y	Y	Y	Y	Y	Y	N/A	Y	Y	Y	Y	Y	20
Gemmell 2016	Y	Y	Y	Y	Y	Y	Y	Y	N	Y	Y	Y	Y	Y	Y	Y	N/A	Y	Y	Y	Y	N	19
Ghahramani 2021	Y	Y	Y	Y	Y	Y	Y	Y	Y	Y	Y	Y	Y	N	Y	Y	N/A	Y	Y	Y	Y	Y	20
Grams 2021	Y	Y	Y	Y	Y	Y	Y	Y	N	N	Y	N	N	Y	Y	Y	N	Y	Y	Y	N	Y	21

	STROBE Item Number																						Total number
Citation	1	2	3	4	5	6	7	8	9	10	11	12	13	14	15	16	17	18	19	20	21	22	
Grandy 2021	N	Y	Y	N	Y	Y	Y	Y	Y	N	Y	Y	Y	Y	Y	N	NA	Y	Y	Y	Y	Y	17
Hall 2018	Y	Y	Y	Y	Y	Y	Y	Y	N	N	Y	Y	Y	Y	Y	Y	N/A	Y	Y	Y	Y	Y	19
Hall 2019	Y	Y	Y	Y	Y	Y	Y	Y	N	Y	Y	Y	Y	Y	Y	Y	Y	Y	Y	Y	Y	Y	21
Harhay 2020	Y	Y	Y	Y	Y	Y	Y	Y	N	Y	Y	Y	Y	Y	Y	Y	Y	Y	Y	Y	Y	Y	21
Hogan 2017	Y	Y	Y	Y	Y	N	Y	Y	N	Y	Y	N	Y	Y	Y	N	N/A	Y	Y	Y	Y	Y	17
Hynes 2019	Y	Y	Y	Y	Y	Y	Y	Y	N	Y	Y	Y	Y	Y	Y	Y	Y	Y	Y	Y	Y	Y	21
Kalantar 2019	Y	Y	Y	Y	Y	Y	Y	Y	Y	Y	Y	Y	N	Y	Y	Y	Y	Y	Y	Y	Y	Y	21
Kim 2024	Y	Y	Y	Y	Y	Y	Y	Y	Y	N	Y	Y	Y	Y	Y	Y	Y	Y	N	Y	N	Y	19
Kharat 2020	Y	Y	Y	Y	Y	Y	Y	Y	Y	Y	Y	Y	Y	Y	Y	Y	N/A	Y	Y	Y	Y	Y	21
Li 2016	Y	Y	Y	Y	Y	Y	Y	Y	Y	Y	Y	Y	N	N	Y	Y	Y	Y	Y	Y	Y	N	19
Liebman 2016	Y	Y	Y	Y	Y	Y	Y	Y	Y	Y	Y	Y	Y	Y	Y	Y	N/A	Y	Y	Y	Y	Y	21
Loor 2024	Y	Y	Y	Y	Y	Y	Y	Y	N	N	Y	Y	Y	Y	Y	Y	Y	Y	Y	Y	Y	Y	20
Lorenz 2017	Y	Y	Y	Y	Y	Y	Y	Y	N	Y	Y	Y	Y	Y	Y	N	N/A	Y	Y	Y	Y	Y	19
Luo 2017	Y	Y	Y	Y	Y	Y	Y	Y	N	Y	Y	Y	N	Y	Y	N	N/A	Y	Y	Y	Y	Y	18
McAdams-Delmarco 2018	Y	Y	Y	Y	Y	Y	Y	Y	N	Y	Y	N	N	Y	Y	Y	Y	Y	Y	Y	Y	Y	19
Meraya 2020	Y	Y	Y	Y	Y	Y	Y	Y	Y	Y	Y	Y	Y	Y	Y	Y	Y	Y	Y	Y	Y	Y	22
Michalopoulos 2022	Y	Y	Y	Y	Y	Y	N	Y	N	Y	Y	Y	Y	Y	Y	Y	Y	Y	Y	Y	Y	Y	20
Mour 2023*	Y	Y	Y	Y	Y	Y	N	N	N	N	N	N	N	Y	Y	Y	N	Y	N	Y	N	N	11
Naljayan 2023	N	Y	Y	Y	Y	Y	N	N	N	Y	Y	Y	Y	Y	N	Y	NA	Y	N	N	Y	NA	13
Ozieh 2019	Y	Y	Y	Y	Y	Y	Y	Y	Y	Y	Y	Y	Y	Y	Y	Y	Y	Y	Y	Y	Y	Y	22
Peipert 2020	Y	Y	Y	Y	Y	Y	Y	Y	N	Y	Y	Y	Y	Y	Y	Y	Y	Y	Y	Y	Y	Y	21
Peipert 2019	Y	Y	Y	Y	N	Y	Y	Y	N	Y	Y	Y	Y	Y	Y	Y	Y	Y	Y	Y	Y	Y	20
Pollock 2025	Y	Y	Y	Y	Y	Y	Y	Y	N	N	Y	N	N	Y	Y	Y	Y	Y	Y	Y	Y	Y	18
Raspovic 2017	Y	Y	Y	Y	Y	N	Y	Y	Y	Y	Y	Y	N	Y	Y	N	N/A	Y	Y	Y	N	Y	17
Reyes 2021	Y	Y	Y	Y	Y	N	Y	Y	N	Y	Y	N	Y	Y	Y	Y	Y	Y	Y	Y	Y	N	18
Rhee 2017	Y	Y	Y	Y	Y	Y	Y	Y	Y	Y	Y	Y	Y	Y	Y	Y	Y	Y	Y	Y	Y	Y	22
Robiner 2021	Y	Y	Y	Y	Y	Y	Y	Y	N	Y	Y	Y	N	Y	Y	Y	N/A	Y	Y	Y	Y	Y	19
Robiner 2022	N	Y	Y	N	N	N	N	Y	N	N	Y	N	Y	Y	Y	N	N/A	Y	Y	Y	Y	Y	12
Small 2017	Y	Y	Y	Y	Y	Y	Y	Y	N	Y	Y	Y	Y	Y	Y	Y	N/A	Y	Y	Y	Y	Y	20
Song 2018	Y	Y	Y	Y	Y	Y	Y	Y	N	Y	Y	Y	N	Y	Y	N	Y	Y	Y	Y	Y	Y	19
Speyer 2024	Y	Y	Y	Y	Y	Y	Y	Y	Y	N	Y	Y	Y	Y	Y	Y	Y	Y	Y	Y	Y	Y	21

	STROBE Item Number																						Total number
Citation	1	2	3	4	5	6	7	8	9	10	11	12	13	14	15	16	17	18	19	20	21	22	
Taylor 2021	Y	Y	Y	Y	Y	Y	Y	Y	N	N	Y	Y	N	Y	Y	Y	Y	Y	N	Y	Y	Y	18
Van Haalen 2020	Y	Y	Y	Y	Y	Y	N	Y	Y	Y	Y	Y	Y	Y	Y	Y	NA	Y	Y	Y	Y	Y	20
Warsame 2018	Y	Y	Y	Y	Y	Y	Y	Y	N	Y	Y	Y	N	Y	Y	N	Y	Y	Y	Y	Y	Y	19
Whittington 2025*	Y	Y	Y	Y	Y	Y	Y	Y	N	N	Y	N	N	Y	Y	N	N	Y	N	Y	N	Y	14

*Conference abstract

Supplemental Table 5. Study characteristics: Patient burden SLR

Author, Year	Geographic region	Study design	Study period	Patient population	N
Abedeen 2023 *(abstract)*	New York, US (inner city)	Cross-sectional	NR	ESKD, dialysis; transplant recipients	40
Acquah 2021[1]	US	Cross-sectional survey (NHIS)	2014-2018	CKD; aged 18-64 yrs	1,425
Adjeroh 2023	US	Cross-sectional	2010-2019	CKD; non-dialysis	649
Awan 2020[2]	US	Retrospective (ESKD registry)	2007-2014	Dialysis initiation; aged 18-54 yrs	75,700
Becker 2020[3]	US	Prospective observational	2018-2017	ESKD	2,545
Borshchenko 2019[4] *(abstract)*	New York, US	Retrospective observational	2014-2018	HD patients	249
Brown 2021[5]	US	Prospective observational ([P]DOPPS)	2012-2019	HD patients	1,441
				PD patients	1,445
Cabacungan 2019[7] *(abstract)*	North Carolina	Cross-sectional	NR	African American patients; kidney transplant waiting list	300
Cervantes 2021[6]	Colorado, US	Cross-sectional	NR	Scheduled HD, after emergency HD (undocumented immigrants)	30
Chadban 2023[8] *(abstract)*	US	Cross-sectional (PACE-CKD)	2022	CKD; aged ≥18 years	199
Cohen 2019[9]	US	Retrospective observational	2014-2016	Dialysis patients	240,343
Daniel 2021[10]	Alabama, US	Cross-sectional	NR	HD patients	124
Das 2024[11] *(abstract)*	US	Retrospective observational (MEPS)	2018-2021	CKD patients	141
Domenick Sridharan 2018[12]	Pittsburgh, US	Prospective observational		HD patients	77

Author, Year	Geographic region	Study design	Study period	Patient population	N
Egeolu 2023	Atlanta, US	Cross-sectional	2021-2022	ESKD + diabetes, with or without diabetic retinopathy	93
Eneanya 2019[13]	US	Retrospective observational	2013-2015	HD and PD patients	5,114
Gemmell 2016[14]	Pittsburgh, US	Prospective observational	2004-2009	CKD	182
Ghahramani 2021[15, 16]	Pennsylvania, US	Prospective observational	NR	CKD, stage 4/5	155
Grams 2021[17]	US	Prospective observational (CRIC)	2003-2018	CKD	3,939
Grandy 2021[18]	US	Retrospective observational	2015, 2017-2018	CKD, stage 3-4, with and without hyperkalaemia	376
Hall 2018[19]	US	Prospective observational	2012-2013	HD; ≥75 yrs	3,132
Hall 2019[20]	US	Prospective observational	2012-2013	Dialysis; ≥65 yrs	1,371
Harhay 2020[21]	US	Prospective observational (CRIC)	2003-2008	Advanced CKD	1,676
Hogan 2017[22]	Dallas, US	Prospective observational	2013	Emergent dialysis in ESKD	88
Hynes 2019[23]	Chicago, US	Prospective observational	2015-2016	ESKD	175
Kalantar 2019[24]	US	Prospective observational	2011-2016	Dialysis	753
Kharat 2020[25]	US (MEPS)	Cross-sectional	2002-2016	CKD + diabetes	3,489
Kim 2024	US	Cross-sectional	2018	Kidney failure	40
Li 2016[26]	Los Angeles, US	Cross-sectional	NR	HD patients	72
Liebman 2016[27]	US	Retrospective observational	2006	HD patients	11,756
Loor 2024	Pittsburg, US	Prospective observational	2010-2018	KT recipients, pre- and post-transplant	166

Author, Year	Geographic region	Study design	Study period	Patient population	N
Lorenz 2017[28]	Rochester, US	Prospective observational	2012-2014	LDKT recipients	140
Luo 2018[29]	US	Retrospective observational	2015-2016	ESKD	11,849
McAdams-DeMarco 2018[30]	Baltimore, US	Prospective observational	2014-2017	KT recipient	443
Meraya 2020	US (MEPS)	Cross-sectional	2009-2015	CKD + diabetes	942
Michalopoulos 2022[31, 32]	US	Cross-sectional, online survey	2020	CKD with and without anemia; aged ≥18 years	410
Mour 2023 *(abstract)*	US	Cross-sectional / prospective observational	2022	ESKD, elderly, pre- and post-KT	128
Nalijayan 2020[33] *(abstract)*	US	Prospective observational	2015-2019	PD (incremental, full)	NR
Naljayan 2023	US	Retrospective observational	2015-2019	PD (CAPD, APD; incremental, full)	1,658
Ozieh 2019[34]	US	Retrospective observational (MEPS-HC data)	2002-2011	Kidney disease, aged 18-64 yrs	2,966
Peipert 2019[35]	US	Prospective observational	NR	Dialysis patients	58,851
Peipert 2020[36]	US	Prospective observational	2007-2016	LDKT recipients	477
Pollock 2025	US	Prospective observational	2019 (baseline)	CKD patients	NR
Raspovic 2017[37]	Dallas, US	Prospective observational	NR	ESKD + diabetes	30
Reyes 2021[38]	New York, US	Cross-sectional	NR	ESKD, outpatient HD	95
Rhee 2017[39]	California, US	Prospective observational	2013-2015	HD patients	450
Robiner 2021[40]	Minnesota, US	Prospective observational	NR	Transplant recipients	28

Author, Year	Geographic region	Study design	Study period	Patient population	N
Robiner 2022	US	Prospective observational	NR	ESKD, KT candidates	139
Small 2017[41]	US	Cross-sectional (MEPS data)	2011-2013	Non-dialysis dependent CKD, aged ≥21 yrs	52
Song 2018[42]	North Carolina, US	Prospective observational	2012-2015	Dialysis patients	277
Speyer 2024	US	Cross-sectional	2013-2021	CKD, stage 3-5, non-dialysis	1,184
Taylor 2021	US	Prospective observational	2014-2020	KT candidates	1,298
Van Haalen 2020[43]	US	Cross-sectional	2012-2018	CKD patients	1,933
Warsame 2018[44]	Baltimore, US	Cross-sectional	2016-2017	HD patients	431
Whittington 2025[45] *(abstract)*	North Carolina, US	Prospective observational	NR	KT patients[1]	10

Abbreviations: CKD=chronic kidney disease; ESKD=end-stage kidney disease; HD=hemodialysis; KT=kidney transplant; LDKT=living donor kidney transplant; MEPS-HC=Medical Expenditure Panel Survey Household Component; NHIS=National Health Interview Survey; PD=peritoneal dialysis; NR=Not reported; US=United States.
1 Kidney transplant caregiver-recipient dyads.

Supplemental Table 6. Patient characteristics: Patient burden SLR

Author, Year	Patient population	N	Mean (SD) age, years	n (%) male	Ethnicity/race	Insurance, n (%)	Employment, n (%)	Income level, n (%)
Abedeen 2023 *(abstract)*	ESKD, dialysis; transplant recipients	40	59.7 (15.6)	26 (70)	African American: 30 (77)	Insured: 40 (100)<br>Uninsured: 0 (0)	NR	<$40,000: 13 (32.5)
Acquah 2021	CKD; aged 18-64 yrs	1,425	48.6 (12.6)	568 (42.5)	Non-Hispanic white: 833 (58.6)<br>Non-Hispanic Black: 238 (14.9)<br>Hispanic: 250 (20.2)<br>Other, non-Hispanic:104 (6.3)	Private: 546 (43.3)<br>Medicaid: 554 (36.1)<br>Uninsured: 185 (12.3)<br>Other: 139 (8.3)	NR	Low income: 786 (49.9)<br>Middle/high income: 580 (50.1)
Adjeroh 2023	CKD; non-dialysis	649	61.6 (13.9)	306 (50.3)	White: 289 (62.9)<br>African American: 211 (20.7)<br>Non-white Hispanic: 102 (10.3)<br>Other: 47 (6.1)	NR	NR	Low income: 343 (44.3)<br>Middle income: 159 (25.7)<br>High income: 147 (30.0)
Awan 2020	ESKD; aged 18-54 years	75,700	NR[1]	48,798 (64.4)	White: 44,209 (58.4)<br>Black: 25,359 (33.5)<br>Other: 6,132 (8.1)	Medicare disability: 4996 (6.6)	Employed: 46,934 (62.0)	NR
Becker 2020	ESKD	2,545	57.2 (12.9)	1,547 (60.8)	White: 399 (15.7)<br>Black: 537 (21.1)<br>Hispanic: 1352 (53.1)<br>Asian: 147 (5.8)<br>Other/missing: 110 (4.3)	NR	NR	NR
Borshchenko 2019 *(abstract)*	HD	249	Median: 62	149 (60)	White: 115 (46)	Private: 62 (25)<br>Medicare:149 (60)<br>Medicaid: 37 (15)	NR	NR
Brown 2021	HD	1,441	62.9 (14.8)	770 (53)	NR	NR	Employed: 42%	NR
	PD	1,445	59.6 (14.8)	796 (55)	NR	NR	Employed: 20%	NR
Cabacungan 2019	African American patients; kidney transplant waiting list	300	52 (11)	168 (56)	African American: 300 (100)	NR	Retired due to disability: 150 (50.0)	Near or below poverty: 75 (25)<br>Household income <$40,000: 129 (43)
Cervantes 2021	Scheduled HD, after emergency HD	30	51.7 (10.6)	20 (66.7)	NR<br>(All undocumented immigrants)	[0 (0)]	Employed: 8 (27)	Income ≤$15,000: 28 (83)

Author, Year	Patient population	N	Mean (SD) age, years	n (%) male	Ethnicity/race	Insurance, n (%)	Employment, n (%)	Income level, n (%)
	(undocumented immigrants)							
Chadban 2023 *(abstract)*	CKD	199	Median: 58	NR	NR	NR	NR	NR
Cohen 2019	Dialysis	240,343	60.7 (14.7)	183,048 (55.4)	White: 129,909 (39.3)<br>Black: 118,141 (35.8)<br>Hispanic: 57,911 (17.5)<br>Asian: 11,473 (3.5)<br>Other/unknown: 12,968 (3.9)	NR	NR	NR
Daniel 2021	HD	124	57.9 (14.3)	60 (48.4)	Caucasian: 27 (21.8)<br>African-American: 97 (78.2)	NR	NR	NR
Das 2024 (abstract)	CKD	141	NR	NR	NR	NR	NR	NR
Domenick Sridharan 2018	HD	77	61.8 (15.9)	43 (55.8)	White: 30 (39.0)<br>Black/African-American: 46 (59.7)<br>Other: 1 (1.3)	NR	NR	NR
Egeolu 2023	ESKD + diabetes, no diabetic retinopathy	23	62.9 (14)	11 (48)	NR	Private: 8 (35)<br>Public: 19 (83)	Employed: 1 (4.5)<br>Disabled: 14 (64)<br>Retired: 7 (32)	<$20,000: 6 (27)<br>$20,000-$50,000: 7 (23)<br>>$50,000: 2 (8)
	ESKD + diabetes, with diabetic retinopathy	70	57.8 (11)	35 (50)	HR	Private: 23 (33)<br>Public: 63 (90)	Employed: 13 (18)<br>Disabled: 36 (51)<br>Retired: 16 (23)	<$20,000: 24 (36)<br>$20,000-$50,000: 15 (23)<br>>$50,000: 8 (11
Eneanya 2019	HD and PD	5,114	60.3 (14)	2864 (56)	Black: 1176 (23)<br>Hispanic: 563 (11)	NR	NR	Mean household income: $50,888
Gemmell 2016	CKD	182	53.0 (14.5)	116 (63.7)	White: 119 (65.4)<br>Black: 57 (31.3)<br>Other: 6 (3.3)	NR	NR	NR
Ghahramani 2021	CKD, stage 4/5	155	NR[1]	88 (56.8)	White: 74 (47.7)<br>Hispanic: 17 (11.0)	NR	Employed: 46 (29.7)	NR
Grams 2021	CKD	3,939	57.7 (11.0)	2161 (54.9)	Black: 1658 (42.1)<br>Hispanic: 497 (12.6)	NR	NR	NR

Author, Year	Patient population	N	Mean (SD) age, years	n (%) male	Ethnicity/race	Insurance, n (%)	Employment, n (%)	Income level, n (%)
Grandy 2021	CKD, stage 3/4, no hyperkalaemia	312	66 (12.6)	179 (57.4)	White: 213 (68.3)<br>Black: 52 (16.7)<br>Non-white Hispanic: 23 (7.4)	NR	Employed FT: 82 (26.8)<br>Employed PT: 25 (8.2)<br>Unemployed: 11 (3.6)	NR
	CKD, stage 3/4, with hyperkalaemia	64	67.3 (13.1)	37 (57.8)	White: 42 (65.6)<br>Black: 12 (18.8)<br>Non-white Hispanic: 6 (9.4)	NR	Employed FT: 14 (22.2)<br>Employed PT: 8 (12.7)<br>Unemployed: 2 (3.2)	NR
Hall 2018	HD; ≥75 yrs	3,132	80.5 (4.4)	1570 (50.1)	Caucasian: 1590 (50.8)<br>African American: 893 (28.5)<br>Hispanic: 420 (13.4)<br>Other: 228 (7.3)	Medicaid: 675 (22.9)<br>No Medicaid: 2270 (77.1)	NR	NR
Hall 2019	Dialysis; ≥65 yrs	1,371	79.9 (4.5)	677 (49.4)	White: 675 (49.3)<br>African American (419 (30.6)<br>Hispanic: 178 (13.0)<br>Other: 98 (7.2)	Medicare: 1,350 (98.5)<br>Dual-eligible coverage: 293 (21.4)	NR	NR
Harhay 2020	Advanced CKD	1,676	58.8 (11.4)	874 (52.5)	Hispanic: 300 (18.0)<br>Non-Hispanic Black: 759 (45.6)<br>Non-Hispanic White: 537 (32.3)	Insured: 1,094 (71.2)<br>Not insured: 164 (10.7)<br>Unknown: 279 (18.2)	NR	≤$20,000: 661 (39.7)<br>>$20,000: 752 (45.2)
Hogan 2017	Emergent dialysis in ESKD	88	46.3 (13.5)	51 (63.0)	Hispanic: 86 (98.9)<br>Asian: 1 (1.1)	NR	NR	NR
Hynes 2019	ESKD	175	54.4 (15.0)	97 (55)	African American: 86 (49)<br>Hispanic: 83 (47)<br>White/other: ≤11	Medicare/Medicaid: 146 (83)<br>No Medicare/Medicaid: 29 (17)	Full-time: 14 (8.0)<br>Part-time (15 (9.0)<br>Not employed 143 (82.0)	<$20,000: 108 (68)<br>$20,000-$39,999: 30 (19)<br>≥$40,000: 22 (14)

Author, Year	Patient population	N	Mean (SD) age, years	n (%) male	Ethnicity/race	Insurance, n (%)	Employment, n (%)	Income level, n (%)
							Self-employed: ≤11	
Kalantar 2019	Dialysis	753	55 (14)	437 (58.0)	Hispanic: 368 (49)<br>African American: 239 (32)<br>Other: 146 (19)	Medicare/Medicaid: 581 (77)<br>Private: 82 (11)<br>Other/Unknown: 90 (12)	NR	NR
Kharat 2020	CKD + diabetes	3,489	NR[1]	1,702 (48.8)	Hispanic: 488 (14.0)<br>Non-Hispanic Black: 583 (16.7)<br>Non-Hispanic Asian: 89 (2.6)<br>Other: 2,327 (66.7)	Private: 1,685 (48.3)<br>Public: 1,570 (45.0)<br>Uninsured: 230 (6.6)	NR	Poor: 970 (27.8)<br>Low income: 691 (19.8)<br>Middle income: 991(28.4)<br>High income: 841 (24.1)
Kim 2024	Kidney failure	40	53.03 (11.83)	11 (27.5)	NR	NR	NR	NR
Li 2016	HD patients	72	52 (13)	49 (68)	NR	NR	NR	NR
Liebman 2016	HD patients	11,756	61.1 (14.7)	6,187 (52.6)	White: 6,686 (56.9)<br>Black: 4,399 (37.4)<br>Other: 671 (5.7)<br><br>Hispanic: 1,773 (15.1)<br>Non-Hispanic: 9,983 (84.9)	NR	NR	NR
Loor 2024	KT recipients, pre- and post-transplant	166	50.8 (14.7)	95 (57.2)	Black: 18 (10.8)<br>Non-white Hispanic: 133 (80.1)<br>Other: 15 (9)	Private: 72 (43.4)<br>Public 34 (20.5)	NR	<$50,000: 32 (51.3)
Lorenz 2017	LDKT recipients	140	51.2 (15.1)	86 (61.4)	White: 127 (90.7)	NR	NR	NR
McAdams-DeMarco 2018	KT recipient	443	52.0 (14.1)	278 (62.7)	African-American: 169 (38.2)	NR	NR	NR
Michalopoulos 2022	CKD (overall); aged ≥ 18 years	410	NR[1]<br>≥55 years: 266 (64.9)	170 (41.5)	Caucasian: 308 (75.1)<br>African American: 70 (17.1)<br>Hispanic: 28 (6.8)<br>Asian American: 8 (2.0)<br>Native American / Alaska Native: 14 (3.4)<br>Other/not answered: 6 (1.5)	NR	Employed: 133 (32.4)	NR

Author, Year	Patient population	N	Mean (SD) age, years	n (%) male	Ethnicity/race	Insurance, n (%)	Employment, n (%)	Income level, n (%)
	CKD plus anemia (subgroup); aged ≥ 18 years	190	NR[1]<br>≥55 years: 117 (61.6)	62 (32.6)	Caucasian: 137 (72.1)<br>African American: 40 (21.1)<br>Hispanic: 13 (6.8)<br>Asian American: 3 (1.6)<br>Native American / Alaska Native: 8 (4.2)<br>Other/not answered: 3 (1.6)	NR	Employed: 55 (28.9)	NR
	CKD - no anemia (subgroup); aged ≥ 18 years	220	NR[1]<br>≥55 years: 149 (67.7)	108 (49.1)	Caucasian: 171 (77.7)<br>African American: 30 (13.6)<br>Hispanic: 15 (6.8)<br>Asian American: 5 (2.3)<br>Native American / Alaska Native: 6 (2.7)<br>Other/not answered: 3 (1.4)	NR	Employed: 78 (35.5)	NR
Mour 2023 *(abstract)*	ESKD, elderly, pre- and post-KT	128	70 (4.1)	NR	White: 104 (81)	NR	NR	NR
Nalijayan 2020	PD (incremental, full)	NR	NR	NR	NR	NR	NR	NR
Naljayan 2023	PD (CAPD, incremental)	107	57.1 (13.5)	53 (49.5)	White: 53 (49.5)<br>Black: 22 (20.6)<br>Other: 32 (30)	Private<br>44 (41.1)	NR	NR
	PD (CAPD, full)	107	58.0 (13.5)	62 (58)	White: 61 (57)<br>Black:24 (22.4)<br>Other: 22 (20.6)	Private: 44 (41.1)	NR	NR
	PD (APD, incremental)	722	57.0 (13.5)	477 (66.1)	White: 401 (55.5)<br>Black: 132 (18.3)<br>Other 189 (26.2)	Private: 392 (54.3)	NR	NR
	PD (APD, full)	722	56.9 (13.3)	441 (61.1)	White: 355 (49.2)<br>Black: 182 (25.2)<br>Other: 185 (25.6)	Private: 405 (56.1)	NR	NR

Author, Year	Patient population	N	Mean (SD) age, years	n (%) male	Ethnicity/race	Insurance, n (%)	Employment, n (%)	Income level, n (%)
Ozieh 2019	Kidney disease, aged 18-64 yrs (MEPS-HC)	2,966	NR[1]	(50.9)	Non-Hispanic white: 2,115 (71.3)<br>Non-Hispanic Black: 332 (11.2)<br>Hispanic: 389 (13.1)<br>Other: 131 (4.4)	Private: 2,055 (69.3)<br>Public: 578 (19.5)<br>Uninsured: 332 (11.2)	NR	Poor: 569 (19.2)<br>Low income: 392 (13.2)<br>Middle income: 884 (29.8)<br>High income: 1,115 (37.6)
Pollock 2025	CKD	NR	NR	NR	NR	NR	NR	NR
Raspovic 2017	ESKD + diabetes	30	Median: 59 (46.8-61.0)	23 (76.7)	NR	NR	NR	NR
Reyes 2021	ESKD, outpatient HD	95	58.0 (14.0)	53 (56)	Black: 42 (44)<br>Latino: 38 (40)	NR	NR	<$30,000: 77 (81)
Rhee 2017	HD	450	54.7 (14.5)	243 (54)	Hispanic: (51)<br>Non-Hispanic (49)	Medicare/Medicaid: (77)<br>Private: (11)<br>Other/unknown (12)	NR	NR
Robiner 2021	Transplant recipients	28	50.9 (14.0)	17 (60.7)	African American: 1 (3.6)<br>Asian: 1 (3.6)<br>Caucasian: 25 (89.3)<br>Hispanic: 0 (0)<br>Native American: 1 (3.6)<br>Other: 0 (0)	NR	NR	NR
Robiner 2022	ESKD, KT candidates	139	54.33 (12.69)	80 (57.6)	White: 117 (84.2)<br>Black: 13 (9.4)<br>Non-white Hispanic: 4 (2.9)<br>Asian: 1 (0.7)<br>Native American (3 (2.2)<br>Other: 1 (0.7)	NR	NR	NR
Small 2017	Non-dialysis dependent CKD, aged ≥21 yrs	52	65.5 (NR)	25 (48.7)	Non-Hispanic white: 41 (79.7)<br>Non-Hispanic Black: 6 (11.6)<br>Hispanic: 4 (7.4)<br>Other: 1 (1.3)	Private: 34 (65.2)<br>Public: 14 (26.8)<br>Uninsured: 4 (8.1)	NR	Poor: 5 (9.3)<br>Near Poor: 1 (2.6)<br>Low income: 9 (17.2)<br>Middle income: 17 (32.8)<br>High income: 20 (38.1)

Author, Year	Patient population	N	Mean (SD) age, years	n (%) male	Ethnicity/race	Insurance, n (%)	Employment, n (%)	Income level, n (%)
Song 2018	Dialysis patients	277	58.7 (12.6)	144 (52)	African American: 168 (74.0)<br>White: 52 (22.9)<br>Other: 7 (3.1)		Employed: 18 (7.9)	<$20,000: 119 (52.4)<br>$20,000-$49,999: (73 (32.2)<br>≥$50,000: 26 (11.5)
Speyer 2024	CKD, stage 3-5, non-dialysis	1,184	69.5 (12.0)	601 (50.8)	NR	NR	NR	NR
Taylor 2021	KT candidates; low disease burden	420	56 (13)	374 (65)	White: 187 (45)<br>Black: 189 (45)<br>Non-white Hispanic: 17 (4)<br>Other (27 (6)	NR	NR	NR
	KT candidates, medium disease burden	403	55 (13)	263 (65)	White: 197 (49)<br>Black: 150 (37)<br>Non-white Hispanic: 16 (4)<br>Other: 40 (10)	NR	NR	NR
	KT candidates, high disease burden	202	55 (13)	104 (51)	White: 99 (49)<br>Black: 84 (42)<br>Non-white Hispanic: 7 (4)<br>Other: 12 (6)	NR	NR	NR
	KT candidates, very high disease burden	273	52 (13)	142 (52)	White: 120 (44)<br>Black: 114 (42)<br>Non-white Hispanic: 16 (6)<br>Other: 23 (8)	NR	NR	NR
Van Haalen 2020	CKD	1,933	59.8 (14.2)	1064 (55)	White/Caucasian: 1063 (55)<br>Chinese: 37 (2)<br>African American: 512 (27)<br>Hispanic/Latino: 197 (10)<br>Asian, other: 64 (3)<br>Other: 59 (3)	NR	Employed: 574 (30)<br>Retired: 719 (37)<br>Other: 558 (29)	NR
Warsame 2018	HD patients	431	54 (13)	279 (64.7)	African American: 231 (53.6)<br>Hispanic: 420 (97.4)	NR	Employed: 111 (25.8)	NR

Author, Year	Patient population	N	Mean (SD) age, years	n (%) male	Ethnicity/race	Insurance, n (%)	Employment, n (%)	Income level, n (%)
Whittington 2025 (*abstract*)	KT patients	10	NR	NR	Black: 3 (30)	NR	NR	NR

Abbreviations: CKD=chronic kidney disease; ESKD=end-stage kidney disease; HD=hemodialysis; MEPS-HC=Medical Expenditure Panel Survey Household Component; NR=Not reported.

[1] These studies did not report mean age,but reported number of patients within defined age bands.

Supplemental Table 7. Study characteristics: Caregiver burden SLR

Author, Year	Geographic region	Study design	Study period	Patient population	N caregivers
Affinito 2018	US	Cross-sectional	NR	ESKD patients receiving hemodialysis	89
Chadban 2023 *(abstract)*	US	Cross-sectional	2022	CKD patients	113
Ghahramani 2022	Pennsylvania, US	Prospective	NR	CKD patients	86
Michalopoulos 2022	US	Cross-sectional	2020	CKD patients with and without anemia	258
Rasmussen 2020	Maryland, US	Prospective	2016-2019	Pre-transplant patients on dialysis; post-transplant patients	99
Starks 2019	Mid-South US	Cross-sectional	NR	Dialysis recipients	75
Whittington 2025 *(abstract)*	North Carolina, US	Prospective observational	NR	KT patients[1]	10

Abbreviations: CG=caregiver; CKD=chronic kidney disease; ESKD=end-stage kidney disease; HD=hemodialysis; NR=Not reported; US=United States.

[1] Kidney transplant caregiver-recipient dyads.

Supplemental Table 8. Patient characteristics: Caregiver burden SLR

Author, Year	Patient population	N	Mean (SD) age, years	n (%) female	Ethnicity/race, n (%)
Affinito 2018	ESKD patients on HD; aged ≥ 18 years	NR	NR	NR	NR
Chadban 2023 *(abstract)*	CKD patients; 32% dialysis-dependent (PACE-CKD study)	199	Median: 58 years	NR	NR
Ghahramani 2022	CKD	NR	NR	NR	NR
Michalopoulos 2022	CKD (overall); aged ≥ 18 years	258	NR[1] ≥55 years, n (%): 182 (70.5)	120 (46.5)	Caucasian: 187 (72.5) African American: 32 (12.4) Hispanic: 32 (12.4) Asian American: 15 (5.8) Native American / Alaska Native: 10 (3.9) Other/not answered: 1 (0.4)
	CKD plus anemia (subgroup); aged ≥ 18 years	110	NR[1] ≥55 years, n (%): 69 (62.7)	59 (53.6)	Caucasian: 71 (64.5) African American: 20 (18.2) Hispanic: 15 (13.6) Asian American: 3 (2.7) Native American / Alaska Native: 4 (3.6) Other/not answered: 1 (0.9)
	CKD - no anemia (subgroup); aged ≥ 18 years	148	NR[1] ≥55 years, n (%): 113 (76.4)	61 (41.2)	Caucasian: 116 (78.4) African American: 12 (8.1) Hispanic: 17 (11.5) Asian American: 5 (3.4) Native American / Alaska Native: 6 (4.1) Other/not answered: 0 (0.0)
Rasmussen 2020	Pre-transplant patients on dialysis; post-transplant patients	NR	NR	NR	NR

Author, Year	Patient population	N	Mean (SD) age, years	n (%) female	Ethnicity/race, n (%)
Starks 2019	Dialysis recipients	NR	NR	NR	NR
Whittington 2025 *(abstract)*	KT patients[2]	10	NR	5 (50)	African American: 3 (30)

Abbreviations: CKD=chronic kidney disease; ESKD=end-stage kidney disease; HD=hemodialysis; NR=Not reported.
[1] These studies did not report mean age, but reported number of patients within defined age bands.
[2] Kidney transplant caregiver-recipient dyads.

Supplemental Table 9. Caregiver characteristics: Caregiver burden SLR

Author, Year	Caregiver description	N	Mean (SD) age, years	n (%) female	Ethnicity/race	Employment status, n (%)	Relationship with patient (providing care to), n (%)
Affinito 2018	Primary unpaid CG; aged ≥ 18 years	89	58.5 (13.6)<br>Median: 61<br>(range: 22-91)	85 (95.5)	Caucasian: 62 (70.5)<br>African American: 15 (17.0)<br>Hispanic: 7 (8.0)<br>Asian American: 1 (1.1)<br>Other: 3 (3.4)	Not employed: 32 (37.6)<br>Retired: 6 (7.1)<br>Employed, part-time: 15 (17.6)<br>Employed, full-time: 32 (32.7)	Spouse/partner: 60 (69.0)<br>Child: 3 (3.4)<br>Parent: 18 (20.6)<br>Sibling: 1 (1.1)<br>Other: 5 (5.7)
Chadban 2023 *(abstract)*	CG; unpaid (CKD pt)	113	Median: 38 years	NR	NR	NR	Parent: 51 (45.1)<br>Spouse/partner: 29 (25.7)
Ghahramani 2022	CG; aged ≥ 18 years; undergoing peer-led mentoring	86	NR[1]	55 (64.0)	Caucasian: 40 (47)<br>Hispanic: 8 (9)	Employed: 36 (42)	NR
Michalopoulos 2022	CG; aged ≥ 18 years (CKD pts)	258	NR[1]<br><55 years: 152 (58.9)	196 (76.0)	Caucasian: 199 (77.1)<br>African American: 31 (12.0)<br>Hispanic: 30 (11.6)<br>Asian American: 9 (3.5)<br>Native American / Alaska Native: 6 (2.3)<br>Other/not answered: 2 (0.8)	Employed: 141 (54.7)	Spouse/partner: 103 (39.9)<br>Child: 21 (8.1)<br>Parent: 68 (26.4)<br>Sibling: 9 (4.7)<br>Other relative: 19 (7.4)<br>Friend/neighbor: 24 (9.3)<br>Other/unknown: 5 (1.9)
	CG; aged ≥ 18 years (CKD plus anemia pts)	110	NR[1]<br><55 years: 76 (69.1)	80 (72.7)	Caucasian: 79 (71.8)<br>African American: 18 (16.4)<br>Hispanic: 15 (13.6)<br>Asian American: 6 (5.5)<br>Native American / Alaska Native:	Employed: 67 (60.9)	Spouse/partner: 36 (32.7)<br>Child: 5 (4.5)<br>Parent: 32 (29.1)<br>Sibling: 3 (2.7)<br>Other relative: 13 (11.8)<br>Friend/neighbor: 18 (16.4)

Author, Year	Caregiver description	N	Mean (SD) age, years	n (%) female	Ethnicity/race	Employment status, n (%)	Relationship with patient (providing care to), n (%)
					3 (2.7)<br>Other/not answered: 2 (1.8)		Other/unknown: 3 (2.7)
	CG; aged ≥ 18 years (CKD - no anemia pts)	148	NR[1]<br>≥55 years: 76 (51.4)	116 (78.4)	Caucasian: 120 (81.1)<br>African American: 13 (8.8)<br>Hispanic: 15 (10.1)<br>Asian American: 3 (2.0)<br>Native American / Alaska Native: 3 (2.0)<br>Other/not answered: 0 (0.0)	Employed: 74 (50.0)	Spouse/partner: 67 (45.3)<br>Child: 16 (10.8)<br>Parent: 36 (24.3)<br>Sibling: 9 (6.1)<br>Other relative: 6 (4.1)<br>Friend/neighbor: 12 (8.1)<br>Other/unknown: 2 (1.4)
Rasmussen 2020	CG, spouse or partner (All patients)	99	Median (IQR): 59 (49-66)	67 (77.1)	Caucasian: 48 (55.1)<br>African American: 34 (39.1)<br>Hispanic: 1 (1.2)<br>Asian American: 1 (1.2)<br>Native American / Alaska Native: 1 (1.2)<br>Other/not answered: 3 (3.5)	NR	Spouse/partner: 99 (100)
	CG, spouse or partner (Pre-transplant patients)	65	Median (IQR): 60 (50-65)	47 (77.1)	Caucasian: 32 (51.6)<br>African American: 25 (40.3)<br>Hispanic: 0 (0.0)<br>Asian American: 1 (1.6)<br>Native American / Alaska Native: 1 (1.6)<br>Other/not answered: 3 (4.8)	NR	Spouse/partner: 100%
	CG, spouse or partner (Post-transplant patients)	34	Median (IQR): 57 (45-67)	20 (80)	Caucasian: 16 (64.0)<br>African American: 9 (36.0)<br>Hispanic: 1 (4.0)<br>Asian American: 0 (0.0)<br>Native American / Alaska Native: 0 (0.0)<br>Other/not answered: 0 (0.0)	NR	Spouse/partner: 100%
Starks 2019	CG, aged ≥ 18 years, African American women	75	47 (14)	75 (100)	African American: 75 (100)	Employed, full-time: 33 (44)<br>NR: 42 (56)	Spouse: 18 (24)<br>Child: 21 (28)<br>NR: 36 (48)
Whittington 2025 *(abstract)*	CG, aged ≥ 18 years[2]	10	NR	7 (70)	African American: 3 (30)	NR	Spouse: 7 (70)

Abbreviations: CG=Caregiver; IQR=inter-quartile range; NR=not reported; SD=standard deviation.

Notes:

[1] These studies did not report mean age, but reported number of patients within defined age bands.

[2] Kidney transplant caregiver-recipient dyads.

Supplemental Table 10. KDQOL-36 scores among individuals with CKD

Author, Year	Patient description	n	BKD[1] Mean (SD)	SPKD[1] Mean (SD)	EKD[1] Mean (SD)	SF-12 MCS[2] Mean (SD)	SF-12 PCS[2] Mean (SD)
Becker 2020	ESKD	2,545	NR	NR	NR	50 (NR)	38 (NR)
Borshchenko 2019 *(abstract)*	HD, ambulance travel to HD	249	NR	NR	NR	48.4 (10.4)	NR
	HD, taxi ride to HD		NR	NR	NR	49.8 (9.5)	NR
	HD, self/family ride to HD		NR	NR	NR	52.5 (9.6)	NR
Cervantes 2021	Emergent/ unscheduled HD (Pre-maintenance HD)	30	27.4 (19.5)	67.9 (20.9)	59.0 (21.4)	49.1 (10.4)	34.9 (10.3)
	Maintenance dialysis (HD)	30	59.1 (33.6)	86.1 (11.9)	83.5 (77.4)	55.3 (9.1)	42.2 (10.0)
Cohen 2019	Dialysis	240,343	51.3 (29.8)	78.1 (16.7)	73.0 (22.7)	49.0 (13.4)	36.6 (12.2)
Eneanya 2019	Dialysis (Day 0-120 after initiation)	5114	54.0 (28)	81.3 (14.1)	77.2 (19.8)	51.7 (10)	38.6 (10.4)
	Dialysis (Day 365-485 after initiation)		54.6 (28.5)	80.7 (14.3)	77.6 (19.8)	52 (9.9)	38.7 (10.7)
Grams 2021	CKD (CRIC)	3,939	82.2 (23.7)	89.1 (15.6)	83.4 (14.8)	50.4 (10.5)	41.3 (11.5)
Hall 2018	HD; ≥75 yrs	3,132	52.6 (29.4)	78.5 (15.6)	74.3 (21.3)	50.9 (10.4)	34.5 (9.9)
Hogan 2017	Emergent dialysis	1,664	25.8 (25.3)	61.6 (18.2)	59.7 (22.1)	42.4 (9.6)	33.2 (8.4)
Hynes 2019	HD	175	46.5	76.5	72.3	49.2	35.5
Mour 2023	KT waitlist	49	50.70 (27.2)	81.20 (14.7)	70.40 (19.3)	NR	
	KT, 4 months post-transplant	35	73.2 (28.5)	89.1 (7.5)	83.4 (19.4)	NR	
	KT, 1 year post-transplant	44	71.4 (29.1)	85.5 (13.7)	81.0 (16.8)	NR	
Naljayan 2020	Incremental PD	NR	60.2	NR	79.4	NR	37.7
	Full PD	NR	45.6	NR	72.3	NR	NR
Peipert 2019	Dialysis		52.8	79	74.1	50.9	37.8
Van Haalen 2020	CKD, stage 3a NDD	205	NR	NR	NR	50.5 (9.4)	45.9 (9.3)
	CKD, stage 3b NDD	245	NR	NR	NR	50.2 (8.7)	44.2 (9.4)
	CKD, stage 4 NDD	350	NR	NR	NR	47.8 (9.6)	39.4 (10.4)
	CKD, stage 5 NDD	20	NR	NR	NR	48.7 (10.2)	38.8 (12.0)
	CKD, all NDD	820	NR	NR	NR	49.2 (9.4)	42.4 (10.3)
	CKD, DD	714	NR	NR	NR	48.2 (9.4)	38.8 (9.6)

Notes:
[1] Decreasing scores indicate greater burden, symptoms, effects.
[2] Decreasing scores indicate worse health
Abbreviations: BKD=Burden of Kidney Disease subscale; CKD=chronic kidney disease; EKD=Effects of Kidney Disease subscale; HD=hemodialysis; HRQoL=health-related quality of life; KDQOL-36=Kidney Disease and Quality of Life-36 survey; MCS=mental component score; NR=not reported; PCS=physical component score; PD=peritoneal dialysis; SPKD=Symptoms and Problems of Kidney Disease subscale.

Supplemental Table 11. KDQOL-SFscores among individuals with CKD

Author, Year	Patient description	n	BKD Mean (SD)	SPKD Mean (SD)	EKD Mean (SD)	SF-12 MCS Mean (SD)	SF-12 PCS Mean (SD)
A							
Brown 2021	HD	1,441	Median: 43.8 (NR)	NR	NR	Median: 49	Median: 34.4
	PD	1,445	Median: 56.3 (NR)	NR	NR	Median: 52	Median: 38.1
Daniel 2021	HD	124	NR	NR	NR	65.3 (22.0)	50.1 (21.7)
Li 2016	HD	72	43.2 (27.6)	79.9 (16.3)	65.0 (25.9)	50.6 (10.2)	40.4 (9.5)
McAdams-DeMarco 2018	Pre-transplant		NR	NR	NR	52.8 (8.9)	43.3 (9.6)
Peipert 2020	Pre-transplant	443	NR	NR	NR	48.6	42.5
Warsame 2018	HD	431	NR	NR	NR	52.8 (10.1)	41.6 (9.9)

Notes:
[1] Decreasing scores indicate greater burden, symptoms, effects.
[2] Decreasing scores indicate worse health
Abbreviations: BKD=Burden of Kidney Disease subscale; CKD=chronic kidney disease; EKD=Effects of Kidney Disease subscale; HD=hemodialysis; HRQoL=health-related quality of life; KDQOL-36=Kidney Disease and Quality of Life-36 survey; MCS=mental component score; NR=not reported; PCS=physical component score; PD=peritoneal dialysis; SPKD=Symptoms and Problems of Kidney Disease subscale.

Supplemental Table 12. EQ-5D scores among individuals with CKD

Author, Year	Patient description	Subgroup	n	VAS Mean (SD)
Grandy 2021	CKD, stage 3/4	No hyperkalaemia	312	69.6 (-)
		With hyperkalaemia	64	65.5 (-)
Van Haalen 2020	CKD	Stage 3a NDD	208	76.3 (15.3)
		Stage 3b NDD	247	73.3 (15.2)
		Stage 4 NDD	350	65.5 (17.5)
		Stage 5 NDD	22	69.8 (18.4)
		All NDD	827	70.7 (16.9)
		DD	754	65.6 (18.4)

Abbreviations: CKD=chronic kidney disease; DD=dialysis dependent; NDD=non-dialysis dependent; SD=standard deviation; VAS=visual analogue scale.

Supplemental Table 13. Summary of caregiver burden scale scores - ZBI

Author, Year	Caregiver description	Overall or subgroup	N	Overall score Mean (SD)
Ghahramani 2022	CG; aged ≥ 18 years; undergoing peer-led mentoring (CKD patients)	Mentoring (face-to-face) Baseline	29	21.1 (9.4)
		Mentoring (face-to-face) 12 months		16.0 (7.6)
		Mentoring (face-to-face) 18 months		14.0 (7.8)
		Mentoring (online) Baseline	29	23.6 (12.1)

Author, Year	Caregiver description	Overall or subgroup	N	Overall score<br>Mean (SD)
		Mentoring (online)<br>12 months		16.5 (9.1)
		Mentoring (online)<br>18 months		15.2 (9.9)
		Mentoring (textbook only)<br>Baseline	28	22.1 (9.3)
		Mentoring (textbook only)<br>12 months		18.6 (9.7)
		Mentoring (textbook only)<br>18 months		22.0 (9.9)
Rasmussen 2020[49]	CG, spouse or partner<br>(All patients)	Overall	99	Median (IQR):<br>18.5 (11-32)
	CG, spouse or partner<br>(Pre-transplant patients)	Pre-transplant	65	Median (IQR):<br>19 (11-35)
	CG, spouse or partner<br>(Post-transplant patients)	Post-transplant	34	Median (IQR):<br>16 (12-24)
Starks 2019[50]	CG, aged ≥ 18 years, African American women<br>(dialysis patients)	Overall	75	18.0 (14.0)

Abbreviations: CG=caregiver; IQR=interquartile range; n=number; SD=standard deviation; ZBI=Zarit Burden Inventory.

Supplemental Table 14. Summary of caregiver burden scale scores – BSFC-s

Author, Year	Caregiver description	Overall or subgroup	N	Outcome description – level of burden	n (%)
Michalopoulos 2022	CG; aged ≥ 18 years (Overall)	CKD plus anemia patients	110	None to low	6 (5.5)
				Moderate	28 (25.5)
				Severe to very severe	76 (69.1)
		CKD - no anemia patients	148	None to low	11 (7.4)
				Moderate	50 (33.8)
				Severe to very severe	87 (58.8)
	CG; aged ≥ 18 years - Living with care recipient	CKD plus anemia patients	56	None to low	3 (5.5)
				Moderate	10 (18.2)
				Severe to very severe	43 (76.4)
		CKD - no anemia patients	90	None to low	8 (9.2)
				Moderate	29 (32.2)
				Severe to very severe	53 (58.6)

Abbreviations: BSFC-s= Burden Scale for Family Caregivers-Short Version; CG=caregiver.

References

1. Acquah I, Valero-Elizondo J, Javed Z, Ibrahim HN, Patel KV, Ryoo Ali HJ, et al. Financial Hardship Among Nonelderly Adults With CKD in the United States. Am J Kidney Dis. 2021;78(5):658-68.
2. Awan AA, Zhao B, Anumudu SJ, Winkelmayer WC, Ho V, Erickson KF. Pre-ESKD Nephrology Care and Employment at the Start of Dialysis. Kidney Int Rep. 2020;5(6):821-30.
3. Becker BN, Luo J, Gray KS, Colson C, Cohen DE, McMurray SD, et al. Associations between enrollment in ESRD special needs plans and outcomes. Journal of the American Society of Nephrology. 2020;31:22.
4. Borshchenko Y, Tharian A, Drakakis J, Masani NN, Khatri M, Grant CD. Hemodialysis transportation, compliance, and quality of life. Journal of the American Society of Nephrology. 2019;30:174-5.
5. Brown EA, Zhao J, McCullough K, Fuller DS, Figueiredo AE, Bieber B, et al. Burden of Kidney Disease, Health-Related Quality of Life, and Employment Among Patients Receiving Peritoneal Dialysis and In-Center Hemodialysis: Findings From the DOPPS Program. Am J Kidney Dis. 2021;78(4):489-500 e1.
6. Cervantes L, Tong A, Camacho C, Collings A, Powe NR. Patient-reported outcomes and experiences in the transition of undocumented patients from emergency to scheduled hemodialysis. Kidney Int. 2021;99(1):198-207.
7. Cabacungan AN, Ellis MJ, Sudan D, Davenport CA, Ephraim P, Strigo TS, et al. Financial strain and pursuit of live donor kidney transplants among african americans on the kidney transplant waiting list. Journal of the American Society of Nephrology. 2019;30:426.
8. Chadban SE, C.; Rangaswami, J.; Wu, M.; Hull, R.; Elsayed, J.; Reichel, H.; Garica Sanchez, JJ.; Pentakota, S.; Kularatne, T.; Fifer, S. . Pace-Ckd: Financial Burden and Work Productivity of Patients with Ckd and Caregivers: Results from a Us Survey. Nephrology Dialysis Transplantation. 2023.
9. Cohen DE, Lee A, Sibbel S, Benner D, Brunelli SM, Tentori F. Use of the KDQOL-36 for assessment of health-related quality of life among dialysis patients in the United States. BMC Nephrol. 2019;20(1):112.
10. Daniel SC, Azuero A, Gutierrez OM, Heaton K. Examining the relationship between nutrition, quality of life, and depression in hemodialysis patients. Qual Life Res. 2021;30(3):759-68.
11. Das AK T, SL, editor Out-of-Pocket Expenditures for Patients with CKD in the Medical Expenditure Panel Survey (MEPS). Journal of the American Society of Nephrology; 2024.
12. Domenick Sridharan N, Fish L, Yu L, Weisbord S, Jhamb M, Makaroun MS, et al. The associations of hemodialysis access type and access satisfaction with health-related quality of life. J Vasc Surg. 2018;67(1):229-35.
13. Eneanya ND, Maddux DW, Reviriego-Mendoza MM, Larkin JW, Usvyat LA, van der Sande FM, et al. Longitudinal patterns of health-related quality of life and dialysis modality: a national cohort study. BMC Nephrol. 2019;20(1):7.
14. Gemmell LA, Terhorst L, Jhamb M, Unruh M, Myaskovsky L, Kester L, et al. Gender and Racial Differences in Stress, Coping, and Health-Related Quality of Life in Chronic Kidney Disease. J Pain Symptom Manage. 2016;52(6):806-12.
15. Lopez EMJ, Ezeji GC, Romeu JC, Bartolomeo K, Chinchilli VM, Ghahramani N. Online peer mentoring and quality of life among patients with CKD. Journal of the American Society of Nephrology. 2019;30:522.
16. Ghahramani N, Chinchilli VM, Kraschnewski JL, Lengerich EJ, Sciamanna CN. Effect of Peer Mentoring on Quality of Life among CKD Patients: Randomized Controlled Trial. Kidney Dis (Basel). 2021;7(4):323-33.

17. Grams ME, Surapaneni A, Appel LJ, Lash JP, Hsu J, Diamantidis CJ, et al. Clinical events and patient-reported outcome measures during CKD progression: findings from the Chronic Renal Insufficiency Cohort Study. Nephrol Dial Transplant. 2021;36(9):1685-93.
18. Grandy S, Jackson J, Moon R, Bluff D, Palaka E. Health-related quality of life and lifestyle changes in patients with chronic kidney disease and hyperkalaemia: Real-world data from the US, five European countries and China. Int J Clin Pract. 2021;75(8):e14326.
19. Hall RK, Luciano A, Pieper C, Colon-Emeric CS. Association of Kidney Disease Quality of Life (KDQOL-36) with mortality and hospitalization in older adults receiving hemodialysis. BMC Nephrol. 2018;19(1):11.
20. Hall RK, Luciano A, Pendergast JF, Colon-Emeric CS. Self-reported Physical Function Decline and Mortality in Older Adults Receiving Hemodialysis. Kidney Med. 2019;1(5):288-95.
21. Harhay MN, Yang W, Sha D, Roy J, Chai B, Fischer MJ, et al. Health-Related Quality of Life, Depressive Symptoms, and Kidney Transplant Access in Advanced CKD: Findings From the Chronic Renal Insufficiency Cohort (CRIC) Study. Kidney Medicine. 2020;2(5):600-9.e1.
22. Hogan AN, Fox WR, Roppolo LP, Suter RE. Emergent dialysis and its impact on quality of life in undocumented patients with end-stage renal disease. Ethnicity and Disease. 2017;27(1):39-44.
23. Hynes DM, Fischer M, Fitzgibbon M, Porter AC, Berbaum M, Schiffer L, et al. Integrating a Medical Home in an Outpatient Dialysis Setting: Effects on Health-Related Quality of Life. Journal of General Internal Medicine. 2019;34(10):2130-40.
24. Kalantar SS, You AS, Norris KC, Nakata T, Novoa A, Juarez K, et al. The Impact of Race and Ethnicity Upon Health-Related Quality of Life and Mortality in Dialysis Patients. Kidney Medicine. 2019;1(5):253-62.
25. Kharat AA, Muzumdar J, Hwang M, Wu W. Assessing trends in medical expenditures and measuring the impact of health-related quality of life on medical expenditures for U.S. adults with diabetes associated chronic kidney disease using 2002-2016 medical expenditure panel survey data. Journal of Pharmaceutical Health Services Research. 2020;11(4):365-73.
26. Li YN, Shapiro B, Kim JC, Zhang M, Porszasz J, Bross R, et al. Association between quality of life and anxiety, depression, physical activity and physical performance in maintenance hemodialysis patients. Chronic Diseases and Translational Medicine. 2016;2(2):110-9.
27. Liebman S, Li NC, Lacson E. Change in quality of life and one-year mortality risk in maintenance dialysis patients. Quality of Life Research. 2016;25(9):2295-306.
28. Lorenz EC, Cheville AL, Amer H, Kotajarvi BR, Stegall MD, Petterson TM, et al. Relationship between pre-transplant physical function and outcomes after kidney transplant. Clinical Transplantation. 2017;31(5) (no pagination)(e12952).
29. Luo J, Lee A, Cohen DE, Colson C, Brunelli SM. Vocational activity and health insurance type among patients with end-stage renal disease: association with outcomes. Journal of Nephrology. 2018;31(4):577-84.
30. McAdams-DeMarco MA, Olorundare IO, Ying H, Warsame F, Haugen CE, Hall R, et al. Frailty and Postkidney Transplant Health-Related Quality of Life. Transplantation. 2018;102(2):291-9.
31. Michalopoulos EN, Gauthier-Loiselle M, Sanon M, Serra E, Bungay R, Clynes D, et al. Reported caregiver burden in CKD with and without anemia: A us-based survey. Journal of the American Society of Nephrology. 2020;31:135.
32. Michalopoulos SN, Gauthier-Loiselle M, Aigbogun MS, Serra E, Bungay R, Clynes D, et al. Patient and Care Partner Burden in CKD Patients With and Without Anemia: A US-Based Survey. Kidney Med. 2022;4(4):100439.

33. Naljayan MV, Tentori F, Hunt A, McKeon KL, Schreiber MJ, Brunelli SM. Use of incremental peritoneal dialysis: Impact on clinical outcomes and quality-of-life measures. Journal of the American Society of Nephrology. 2020;31:429.
34. Ozieh MN, Bishu KG, Dismuke CE, Egede LE. Trends in Out-of-Pocket Burden in United States Adults with Kidney Disease: 2002-2011. American Journal of the Medical Sciences. 2019;358(2):149-58.
35. Peipert JD, Nair D, Klicko K, Schatell DR, Hays RD. Kidney disease quality of life 36-item short form survey (KDQOL-36) normative values for the United States dialysis population and new single summary score. Journal of the American Society of Nephrology. 2019;30(4):654-63.
36. Peipert JD, Caicedo JC, Friedewald JJ, Abecassis MMI, Cella D, Ladner DP, et al. Trends and predictors of multidimensional health-related quality of life after living donor kidney transplantation. Quality of Life Research. 2020;29(9):2355-74.
37. Raspovic KM, Ahn J, La Fontaine J, Lavery LA, Wukich DK. End-Stage Renal Disease Negatively Affects Physical Quality of Life in Patients with Diabetic Foot Complications. International Journal of Lower Extremity Wounds. 2017;16(2):135-42.
38. Reyes M, Fuertes JN, Moore MT, Punnakudiyil GJ, Calvo L, Rubinstein S. Psychological and relational factors in ESRD hemodialysis treatment in an underserved community. Patient Education and Counseling. 2021;104(1):149-54.
39. Rhee CM, Chen Y, You AS, Brunelli SM, Kovesdy CP, Budoff MJ, et al. Thyroid status, quality of life, and mental health in patients on hemodialysis. Clinical Journal of the American Society of Nephrology. 2017;12(8):1274-83.
40. Robiner WN, Petrik ML, Flaherty N, Fossum TA, Freese RL, Nevins TE. Depression, Quantified Medication Adherence, and Quality of Life in Renal Transplant Candidates and Recipients. Journal of Clinical Psychology in Medical Settings. 2021.
41. Small C, Kramer HJ, Griffin KA, Vellanki K, Leehey DJ, Bansal VK, et al. Non-dialysis dependent chronic kidney disease is associated with high total and out-of-pocket healthcare expenditures. BMC Nephrology. 2017;18(1) (no pagination)(3).
42. Song MK, Paul S, Ward SE, Gilet CA, Hladik GA. One-Year Linear Trajectories of Symptoms, Physical Functioning, Cognitive Functioning, Emotional Well-being, and Spiritual Well-being Among Patients Receiving Dialysis. American Journal of Kidney Diseases. 2018;72(2):198-204.
43. Van Haalen H, Jackson J, Spinowitz B, Milligan G, Moon R. Impact of chronic kidney disease and anemia on health-related quality of life and work productivity: Analysis of multinational real-world data. BMC Nephrology. 2020;21(1) (no pagination)(88).
44. Warsame F, Ying H, Haugen CE, Thomas AG, Crews DC, Shafi T, et al. Intradialytic activities and health-related quality of life among hemodialysis patients. American Journal of Nephrology. 2018;48(3):181-9.
45. Whittington OJ, M.; McCracken, E.,; Muench, D.; Russell, G.; Duckworkth, K.; Farney, A.; Orlando, G.; Stratta, R.; Jay C. A Longitudinal Quality of Life Assessment in Kidney Transplant Caregiver-Recipient Dyads. American Journal of Transplantation. 2025.
46. Affinito J, Louie K. Positive Coping and Self-Assessed Levels of Health and Burden in Unpaid Caregivers of Patients with End Stage Renal Disease Receiving Hemodialysis Therapy. Nephrol Nurs J. 2018;45(4):373-9.
47. Abdelhalim A, Khoury AE. Critical appraisal of the top-down approach for vesicoureteral reflux. Investigative and Clinical Urology. 2017;58(Supplement 1):S14-S22.
48. Ezeji GC, Lopez EMJ, Romeu JC, Bartolomeo K, Chinchilli VM, Ghahramani N. Online peer mentoring is associated with improved burden score among caregivers of patients with CKD. Journal of the American Society of Nephrology. 2019;30:520.

49. Van Pilsum Rasmussen SE, Eno A, Bowring MG, Lifshitz R, Garonzik-Wang JM, Al Ammary F, et al. Kidney Dyads: Caregiver Burden and Relationship Strain Among Partners of Dialysis and Transplant Patients. Transplant Direct. 2020;6(7):e566.
50. Starks SA, Graff JC, Wicks MN. Factors Associated With Quality of Life of Family Caregivers of Dialysis Recipients. Western journal of nursing research. 2020;42(3):177-86.
